# Characterization and Functional Analysis of Extracellular Vesicles and Muscle-Abundant miRNAs (miR-1, miR-133a, and miR-206) in C_2_C_12_ Myocytes and *mdx* Mice

**DOI:** 10.1371/journal.pone.0167811

**Published:** 2016-12-15

**Authors:** Yasunari Matsuzaka, Jun Tanihata, Hirofumi Komaki, Akihiko Ishiyama, Yasushi Oya, Urs Rüegg, Shin-ichi Takeda, Kazuo Hashido

**Affiliations:** 1 Administrative Section of Radiation Protection, National Institute of Neuroscience, National Center of Neurology and Psychiatry, Kodaira, Tokyo, Japan; 2 Department of Molecular Therapy, National Institute of Neuroscience, National Center of Neurology and Psychiatry, Kodaira, Tokyo, Japan; 3 Department of Child Neurology, Hospital, National Center of Neurology and Psychiatry, Kodaira, Tokyo, Japan; 4 Department of Neurology, Hospital, National Center of Neurology and Psychiatry, Kodaira, Tokyo, Japan; 5 Department of Pharmacology, Geneva-Lausanne School of Pharmaceutical Sciences, University of Geneva and University of Lausanne, Geneva, Switzerland; Rutgers University Newark, UNITED STATES

## Abstract

Duchenne muscular dystrophy (DMD) is a progressive neuromuscular disorder. Here, we show that the CD63 antigen, which is located on the surface of extracellular vesicles (EVs), is associated with increased levels of muscle-abundant miRNAs, namely myomiRs miR-1, miR-133a, and miR-206, in the sera of DMD patients and *mdx* mice. Furthermore, the release of EVs from the murine myoblast C_2_C_12_ cell line was found to be modulated by intracellular ceramide levels in a Ca^2+^-dependent manner. Next, to investigate the effects of EVs on cell survival, C_2_C_12_ myoblasts and myotubes were cultured with EVs from the sera of *mdx* mice or C_2_C_12_ cells overexpressing myomiRs in presence of cellular stresses. Both the exposure of C_2_C_12_ myoblasts and myotubes to EVs from the serum of *mdx* mice, and the overexpression of miR-133a in C_2_C_12_ cells in presence of cellular stress resulted in a significant decrease in cell death. Finally, to assess whether miRNAs regulate skeletal muscle regeneration *in vivo*, we intraperitoneally injected GW4869 (an inhibitor of exosome secretion) into *mdx* mice for 5 and 10 days. Levels of miRNAs and creatine kinase in the serum of GW4869-treated *mdx* mice were significantly downregulated compared with those of controls. The tibialis anterior muscles of the GW4869-treated *mdx* mice showed a robust decrease in Evans blue dye uptake. Collectively, these results indicate that EVs and myomiRs might protect the skeletal muscle of *mdx* mice from degeneration.

## Introduction

Duchenne muscular dystrophy (DMD; OMIM #310200) is an X-linked recessive, severe and progressive muscle disease with a prevalence of 1 in 3,500 live male births, and is caused by mutations in the *dystrophin* gene [1,2]. This disorder is usually first recognized by muscular weakness from two to five years of age. Subsequently, patients lose their ability to ambulate before 12 years of age, and eventually experience failure of respiratory and cardiac functions owing to degeneration of the diaphragm and cardiac muscles [3]. Serum creatine kinase (CK), which reflects the level of muscle damage, is commonly used as a diagnostic marker for DMD. However, false-positive or false-negative results are commonly observed, because CK release is increased by various factors, such as vigorous exercise [4], and is decreased by disease progression with age following the loss of muscle tissue [5], rendering quantitative diagnosis and prognostic applications of CK difficult. Thus, reliable biomarkers of DMD have been anticipated.

miRNAs are non-coding single stranded RNAs containing approximately 21 to 24 nucleotides, which regulate gene expression by base-pairing of their nucleotides 2 to 8 with the 5′- or 3′-untranslated regions of target mRNAs, primarily in the cytoplasm [6–8]. As the activities of approximately 50% of all coding genes in mammals are predicted to be regulated by miRNAs, the dysregulation of their expressions is associated with the pathophysiological conditions of many disorders. Some miRNAs are encapsulated into microvesicles, exosomes, or apoptotic bodies, whereas other miRNAs form complexes with RNA-binding proteins [9–16]. They can be internalized by recipient cells via extracellular vesicles (EVs), leading to the intercellular communication [13–18]. Despite the high levels of RNase activity within the circulating blood, because miRNAs are protected from RNase by their association with RNA-binding protein(s) or their inclusion within EVs, remarkably stable miRNAs were shown to be secreted into the extracellular space in non-vesicular or vesicular-encapsulated forms [19–21]. Several groups, including our own, previously reported that three myomiRs, namely, miR-1, miR-133a, and miR-206, were increased in the sera of animal models of muscular dystrophy as well as in patients [22–24]. This upregulation in myomiR levels is not limited to DMD patients, as increased levels of miR-1 were found in the sera of Becker muscular dystrophy (BMD), facioscapulohumeral muscular dystrophy, and limb-girdle muscular dystrophy patients, and increased levels of miR-133a and miR-206 were found in BMD patients [25]. In addition, myomiR levels were shown to be inversely correlated with disease severity in DMD patients aged three to six years [22].

The increased levels of myomiRs in the sera of *mdx* mice have been demonstrated to be increased to near wild-type levels by restoration of the dystrophin protein using exon-skipping therapies [26]. On the other hand, in the muscle of *mdx* mice, miR-1 and miR-133a levels have been shown to be downregulated, whereas miR-206 levels are upregulated [26, 27]. The expression of miR-1 and miR-133a in skeletal muscle can be restored by rescue of the dystrophin protein using exon-skipping techniques [27, 28]. These myomiRs have multiple roles in muscle development and regeneration, such as the regulation of genes involved in myogenesis, proliferation, and muscle fiber-type conversion [29, 30]. Thus, miRNAs have been acknowledged not only as noninvasive biomarkers of DMD, but also as therapeutic targets for many disorders. However, the potential involvement of these muscle-abundant miRNAs in the pathogenesis of DMD remains unclear. Elucidating the pathways regulating the release of myomiRs will be valuable towards gaining a better understanding of DMD pathogenesis and new targets for therapies. In fact, the release of miRNAs into intracellular and extracellular spaces was shown to be controlled by the neutral sphingomyelinase 2/ sphingomyelin phosphodiesterase 3 (nSMase2/SMPD3)-regulated secretory machinery of exosomes, which are membrane microvesicles about 30–100 nm in size generated from multivesicular bodies (MVBs) of the terminal endosomal pathway via the biogenesis of ceramide from sphingomyelin [31–35]. However, the mechanisms regulating the functions of myomiRs transported via EVs, including exosomes or larger vesicles, in the pathogenesis of *mdx* mice remain largely unknown. In the present study, we show that the release of EVs from C_2_C_12_ cells is regulated by ceramide metabolism in an intracellular Ca^2+^ concentration-dependent manner. The transport of miR-133a via EVs regulates the viability of C_2_C_12_ cells under conditions of cellular stress. In addition, the inhibition of ceramide synthesis by GW4869 (an inhibitor of exosome secretion), leading to the suppression of EV secretion, reduces muscle degeneration in *mdx* mice. Our findings demonstrate the potential of myomiRs as a novel noninvasive biomarker and the regulation of myomiR levels as a new therapeutic strategy for DMD.

## Materials and Methods

### Antibodies and reagents

Primary antibodies against mouse CD63 (3H1626), rabbit apolipoprotein A-I (apoA-I) (FL-267), and rabbit CD81 (H-121), and the anti-rat IgG- Horseradish peroxidase (HRP) secondary antibody were purchased from Santa Cruz Biotechnology (Santa Cruz, CA). Mouse primary antibody against MHC class II (M5/114.15.2) was purchased from Novus Biologicals (Littleton, CO). Rabbit primary antibody against caveolin-3 was synthesized by Eurofins Genomics K.K. (Tokyo, Japan). Rabbit primary antibody against flotillin-1 was purchased from Bioss USA (Woburn, MA), and the anti-mouse IgG (H+L) HRP secondary antibody was purchased from Bio-Rad Laboratories, Inc. (Berkeley, CA). Anti-rabbit IgG HRP-linked secondary antibody was purchased from Cell Signaling Technology, Inc. (Beverly, MA). Anti-LAMP2 (M3/84) and anti-GAPDH (EPR6256) antibodies were purchased from Abcam (Cambridge, UK). Mouse monoclonal antibody against CD9 (IVA50) was purchased from Abnova (Taipei City, Taiwan). Mouse monoclonal antibody against calnexin (4F10) was purchased from MBL Laboratories CO., LTD (Nagoya, Japan). Mouse monoclonal antibody against HSP90 (H9010) was purchased from StressMarq Biosciences Inc. (Victoria, Canada). Actinomycin D was purchased from Focus Biomolecules (Plymouth Meeting, PA). Methyl-ß-cyclodextrin, 2-aminoethyl diphenylborinate (2-APB), A23187, C6-ceramide, and monensin sodium salt were purchased from Sigma-Aldrich (St. Louis, MO). BAPTA-acetoxymethyl ester and EGTA-acetoxymethyl ester were purchased from DOJINDO Laboratories (Kumamoto, Japan). C2-ceramide and simvastatin were purchased from Focus Biomolecules. Anhydrous caffeine was purchased from Nacalai Tesque (Kyoto, Japan). Cyclopiazonic acid, D-erythro-MAPP, ebselen, nocodazole, and U-0126 were purchased from Cayman Chemical Company (Ann Arbor, MI). GW4869 was purchased from Calbiochem (San Diego, California). Sphingosine-1-phosphate (S1P) was purchased from LKT Laboratories, Inc. (St Paul, MN).

### Patients

Five unrelated Japanese patients with DMD and four healthy controls were enrolled in this study with the approval of the Ethics Committee of the National Center of Neurology and Psychiatry (approval ID: A2011-113), which followed regulation of Helsinki Declaration. All results are treated with standard medical confidentiality and confidential to the extent allowed by law. Written informed consent was obtained from all participants after explaining the details of the study, prior to the collection of peripheral blood.

### Animals

All animals used in this study were housed in a specific pathogen free facility of the National Center of Neurology and Psychiatry and treated in accordance with the guidelines provided by the Ethics Committee for the Treatment of Laboratory Animals of the National Center of Neurology and Psychiatry (approval ID: 2013007), which has adopted the three fundamental principles of replacement, reduction, and refinement. Connsistent with the approvals stipulated by the protocol, all efforts were made to minimize suffering or discomfort to the animals. *mdx* mice with C57BL/6 background, transgenic *mdx* mice overexpressed *dystrophin* gene which lack coding region from exon 45 to exon 55 by CAG promoter with C57BL/6 background (Tanihata J. et al. in preparation of manuscript), and age-matched wild-type C57BL/6 male control mice were used in this study.

### Immunoprecipitation

Two μg of antibodies were mixed with 0.1 mL of sera, and incubated at 4°C overnight. Protein G Sepharose^®^ (100 μL; Sigma-Aldrich) was added to the sera, mixed well, and then incubated at 4°C for 60 min. The immunoprecipitated complexes were collected by centrifugation at 3,000 × g for 2 min at 4°C, and the supernatant was discarded. The pellet was washed with 1 mL of phosphate-buffered saline (PBS). The centrifugation and washing steps were repeated at least 3 times. The pellet was then resuspended in 100 μL of 5% sodium dodecyl sulphate (SDS) solution, heated at 95°C for 5 min, and centrifuged for 1 min at 12,000 × g at room temperature, and then the supernatant was collected as the immunoprecipitated samples.

### Extraction and quantification of miRNA

Total RNA was extracted from 0.1 μL of serum using the mirVana miRNA isolation kit (Ambion, Austin, TX, USA) as 25 μL of RNA eluate according to the manufacturer’s protocol. RNA eluate (4.5 μL) was reverse transcribed using the TaqMan miRNA Reverse Transcription kit (ABI, Foster City, CA) and miRNA-specific stem-loop primers (part of the TaqMan miRNA assay kit, ABI) as previously described (21). The expression levels of miRNA were quantified by real-time PCR using individual miRNA-specific primers (part of TaqMan miRNA assay kit, ABI) with StepOne^™^ Real-Time PCR System (ABI) according to the manufacturer’s protocol. Samples were subjected to real-time PCR in triplicate. The expression of each miRNA was represented as relative values normalized by the expression of miR-16 and cel-miR-39 used as an internal control and spike-in miRNA, respectively. Data analysis was performed by SDS 2.1 real-time PCR data analysis software (ABI). Expression data are shown as median values obtained from three samples together with the standard deviation.

### Purification and quantitation of EVs

Serum was harvested from the peripheral blood of DMD patients in tubes by centrifugation at 3,000 × g for 15 min. Isolation of exosomes from serum was performed by Total Exosome Isolation reagent (Invitrogen) or ExoQuick-LP^™^ (System Bioscience, Palo Alto, CA), according to the manufacturer’s protocol. Briefly, 50 μL of Total Exosome Isolation solution or 63 μL of ExoQuick solution were added to 250 μL of serum. The mixture was vortexed for 15 seconds and then incubated at 4°C for 30 min. After centrifugation at 10,000 × g for 10 min at room temperature, the supernatant was discarded. The centrifugation and aspiration steps were repeated. The pellet containing the EVs was resuspended in 1× PBS. EVs were also isolated from C_2_C_12_ cells, which were conditioned in serum-free media for 24 hr at 90% confluence. Media were harvested, and then centrifuged at 2000 × g for 30 min to remove cells and debris. EVs were then isolated using Total Exosome Isolation reagent (Invitrogen). Briefly, the Total Exosome Isolation reagent (1/5 voulume) was added to cell-free culture media. The solution was vortexed for 15 seconds, and then incubated at 4°C overnight. After centrifugation at 10,000 × g for 1 hour at 4°C, the supernatant was discarded. Again, the centrifugation and aspiration steps were repeated. The pellet containing the EVs was resuspended in 1 × PBS and then filtered through a 0.22 μm filter, Millex-GV (Millipore Corporation, Billerica, MA). The EVs were confirmed using antibodies against exosomal marker proteins as well as flow cytometry. Briefly, recovered exosomes were incubated with 4-μm diameter aldehyde/sulfate latex beads, 4% w/v, (Thermo Fisher Scientific, Waltham, MA) for 15 min at room temperature. The mixture was then mixed in a final volume of 200 μL of PBS at room temperature under gentle agitation. Two hours later, the reaction was terminated by the addition of 100mM glycine for 30 min at room temperature. After centrifugation, the pellet was washed in PBS containing 0.5% of bovine serum albumin and then incubated with specific antibodies. Beads were analyzed by flow cytometry using a BD Biosciences FACSCalibur using FACSDiva version 6.0 software. Concentrations of the EVs were then determined indirectly by quantifying protein concentrations. The amount of released EVs was quantified by measuring the activity of acetylcholinesterase (AChE) (Sigma-Aldrich), as previously described [36]. The total amount of EVs isolated from the culture media was suspended in 800 uL of PBS, and 100 μL of 12.5 mM acetylthiocholine and 100 μL of 1 mM 5,5’-dithio-bis (2-nitrobenzonic acid) (Sigma-Aldrich) was added in a final volume of 1 mL, and then the mixture was incubated at 37°C for 30 min. The EV solution (300 μL each) was divided into 96-well plates in triplicate and then absorbance at 415 nm was measured on a 680 microplate reader (Bio-Rad Laboratories, Inc.).

### Dot blot analysis

Ten μg/μL of EVs extracted from mouse serum was serially diluted in PBS. One μL of the EV solution was dotted onto an Immobilon-P transfer membrane, which is a microporous polyvinylidene fluoride membrane (Millipore Corporation, Billerica, MA). The membrane was dried at 60°C for 10 minutes, and then incubated with primary antibodies (2 ug) at 4°C overnight, and then with HRP-conjugated secondary antibodies at room temperature for 1 hr. Chemiluminescence was detected using ECL Prime Detection Reagent (GE Healthcare, Buckinghamshire, UK) and analyzed using LAS-3000 (Fujifilm Corporation, Tokyo, Japan).

### Western blotting analysis

For Western blotting analysis, 50 μL of sera from *mdx* and wild-type (wt) mice were resolved by 12% SDS PAGE, and then transferred onto a Polyvinylidene Difluoride (PVDF) membrane (Merck Millipore, Billerica, MA). An anti-LAMP2 antibody (1:1,000 in a mixture of tris-buffered saline (TBS) and Polysorbate 20 (TBST)) and an anti-rat IgG-HRP antibody (1:1,000 in TBST) were used as first and secondary antibodies, respectively. Signal detection was performed using antibodies coupled to HRP and ECL Prime Detection Reagent (GE Healthcare, Buckinghamshire, UK), together with analysis by LAS-3000 (Fujifilm Corporation).

### Creatine kinase activity

Serum CK levels were measured using the Fuji Dri-Chem system (Fujifilm Medical Co. Ltd., Tokyo, Japan) according to the manufacturer’s protocol. Serum (10 μL) was deposited on a Fuji Dri-Chem slide and incubated at 37°C. The increase in absorbance by the generated dye was measured spectrophotometrically for 5 min at 540 nm and the activity was calculated according to the installed formula. Data were expressed as units per liter (U/L).

### Cell viability analysis

C_2_C_12_ murine myoblast cells were cultured in Dulbecco's Modified Eagle's medium (DMEM; Sigma-Aldrich) with 10% (v/v) fetal bovine serum (Cell Culture Technologies, Lugano, Switzerland) and 1% (v/v) penicillin/streptomycin (Wako Pure Chemical Industries Ltd., Osaka, Japan) as the growth medium. The cells were grown at 37°C in controlled humidified air with 5% CO_2_. For myogenic differentiation of myoblasts into myotubes, the growth medium was changed to differentiation medium, namely, DMEM containing 2% heat-inactivated horse serum and 1% penicillin/streptomycin. Briefly, C_2_C_12_ cells were grown in 96-well plates (BM Equipment Co. Ltd., Tokyo, Japan) in growth medium until confluent and then changed to serum-free medium (DMEM containing 1% penicillin/streptomycin) and incubated with or without inducers or suppressors of EV secretion for the indicated times. The number of cells was determined by measuring the absorbance at 450 nm of the formazan product in the culture medium using a 680 microplate reader after incubation with 2-(2-methoxy-4-nitrophenyl)-3-(4-nitrophenyl)-5-(2,4-disulfophenyl)-2H-tetrazolium, monosodium salt (WST-8) from Cell Counting Kit-8 (CCK-8) (DOJINDO Laboratories).

### Transfection assay

Three DNA fragments, namely, precursors of miR-1a, miR-133a, and miR-206 (pre-miR-1a, pre-miR-133a, and pre-miR-206) were synthesized (Bioneer, Alameda, CA), and inserted into the intron site of the pEM-157 vector, which contains the *cytomegalovirus* promoter driving transcription of the dsRed-fluorescent protein-coding sequence interrupted by an intron. The sequences of the three fragments were as follows: pre-miR-1a: 5′-GTTTAAACCCAGGCCACATGCTTCTTTATATCCTCATAGATATCTCAGCACTATGGAATGTAAGGAAGTGTGTGGTTTTGGACTAGT-3′, pre-miR-133a: 5′-GTTTAAACAGAAGCCAAATGCTTTGCTGAAGCTGGTAAAATGGAACCAAATCAGCTGTTGGATGGATTTGGTCCCCTTCAACCAGCTGTAGCTGCGCATTGATCACGCCGCAACTAGT-3′, pre-miR-206: 5′-GTTTAAACGCTTGGGACACATACTTCTTTATATGCCCATATGAACCTGCTAAGCTATGGAATGTAAAGAAGTATGTATTTCAGGCACTAGT-3′. C_2_C_12_ cells cultured in 24-well dishes were transfected with these plasmids using Lipofectamine 2000 (Invitrogen) according to the manufacturer’s instructions. Briefly, 0.2 μg of each plasmid was diluted in 25 μL of Opti-MEM^®^I Reduced Serum Medium (Invitrogen). The plasmid solutions were mixed with 4 μL of PLUS^™^ Reagent (Invitrogen), and then incubated at room temperature for 15 min. After incubation, the plasmid solutions were mixed with 25 μL of Lipofectamine 2000, diluted 25 times by Opti-MEM^®^ I Reduced Serum Medum, and then incubated at room temperature for 15 min. The plasmid solutions were added to C_2_C_12_ cells in serum-free medium. After incubation at 37°C for 3 hrs, the medium was changed to growth medium, and then incubated at 37°C for the indicated times.

### Caspase-3 assay

C_2_C_12_ myoblast cells were cultured to confluence in growth medium in 6-well plates, and then the growth medium was changed to serum-free medium (DMEM containing 1% penicillin/streptomycin with 0.5 mM of H_2_O_2_). The cells were incubated with or without EVs (4 μg) for 24 hours. Then, caspase-3 activity was quantified using the Caspase-3/CPP32 Colorimetric Assay Kit (BioVision Inc., Milpitas, CA). Briefly, cells were harvested, and the supernatant was removed by centrifugation. Pellets were suspended in 50 μL of chilled cell lysis buffer, and then incubated on ice for 10 min, and centrifuged for 1 min at 10,000 × g. Supernatants were transferred to fresh tubes, and protein concentrations were measured. Protein solutions were adjusted to a concentration of 100 μg/μL by dilution with PBS. Fifty μL of 2 × Reaction Buffer and 5 μL of 4 mM DEVD-pNA substrate were added to 50 μL of the protein solutions and then incubated at 37°C for 2 hrs. Caspase-3 activity was determined by measuring the absorbance at 405 nm using a 680 microplate reader.

### Analysis of skeletal muscle regeneration of *mdx* mice

GW4869 was dissolved in dimethyl sulfoxide (DMSO) at a concentration of 1.5 mM, and then 2.5 μL of 5% methane sulfonic acid solution was added to 50 μL of the GW4869 solution, resulting in a final concentration of 1.43 mM of GW4869. The solution was then incubated at 37°C until the color of the solution became clear, and the GW4869 solution was further diluted to 100 μM in PBS. One-hundred μL of this final GW4869 solution was intraperitoneally injected into *mdx* mice. After 5 or 10 days, whole body blood was collected from the abdominal aorta. Twenty-four hours before sacrifice, mice were injected intraperitoneally with 1% evans blue dye (EBD; Nacalai Tesque) in PBS as described previously [37]. Muscles were harvested, frozen in melting isopentane, and sectioned. The muscle sections were incubated in ice-cold acetone for 10 min, washed three times for 10 min with PBS, and mounted with Vectashield mounting medium. The presence of EBD in myofibers was analyzed by fluorescence microscopy.

### Secondary structure of myomiRs

GC contents and ΔG were estimated using by a web tool, which is available free of charge at https://sg.idtdna.com/calc/analyzer.

### Statistical analyses

Statistical analysis was performed by the Fisher’s exact probability test (*P*-value test) followed by Bonferroni correction (*P*c). A *P*c-value or *P*-value of less than 0.05 were considered to indicate a statistically significant difference between two groups.

## Results

### Expression levels of myomiRs and EV contents in sera of *mdx* mice and DMD patients

**Fig 1:**
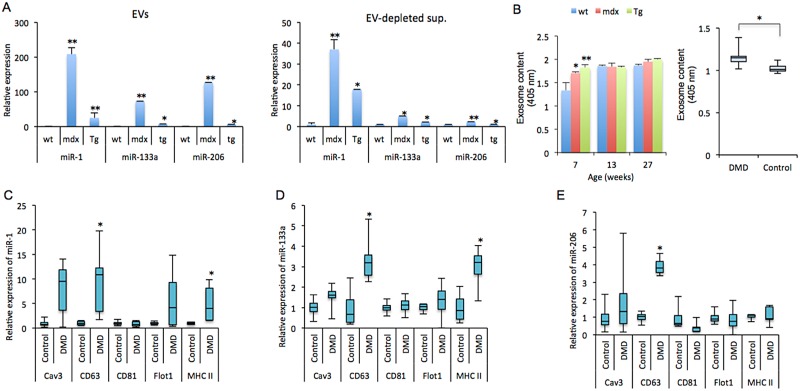
Levels of myomiRs and EVs in the sera of DMD patients and *mdx* mice. (A) Levels of miR-1, miR-133a, and miR-206 in EVs-containing and EV-depleted supernatants, separated from the sera of wt, *mdx*, and *tg* mice (7-weeks old, n = 3, 4, and 4, respectively). Data are represented as means + S.E. *: *P* < 0.05, **: *P* < 0.01 for *mdx* vs wt or *mdx* vs *tg*. (B) EVs were extracted and quantified by AChE activity from the sera of wt, *mdx*, and *tg* mice at 7, 13, and 27 weeks of age (n = 3, 4, and 4, respectively, *: *P* < 0.05, **: *P* < 0.01 vs wt) (left) and DMD patients and healthy controls (right), (n = 5 and 4, respectively, *: *P* < 0.05). (C-E) miR-1 (C), miR-133a (D), and miR-206 (E) levels in EVs separated by immunoprecipitation with anti-caveolin-3 (cav3), anti-CD63, anti-CD81, anti-flotillin-1 (flot1), or anti-MHC class II (MHC II) antibodies in the sera of DMD patients and controls (n = 5 and 4, respectively). *: *P* < 0.05 vs controls.

We previously showed that myomiRs in the serum of mice were upregulated by cardiotoxin-induced muscle injury [25]. It has also been reported that miR-1 and miR-133a levels are downregulated in dystrophic muscle and recover to normal levels by the rescue of dystrophin expression [26, 27]. To elucidate whether myomiR levels in the serum are positively associated with muscle degeneration, the levels of three myomiRs, namely, miR-1, miR-133a, and miR-206, in the sera of transgenic (*tg*) *mdx* mice, overexpressing a truncated dystrophin transcript with a deletion from exon 45 to 55, *mdx* mice, and wild-type (wt) mice were quantified by qRT-PCR. Consistent with a previous report [26], the levels of myomiRs in the serum of *mdx* mice were upregulated compared with those of wt mice, but the *tg* mice showed a decreased level compared with that of *mdx* mice (S1A Fig). Ubiquitously expressed miR-21, mir-29, and small nucleolar RNA 202 (sno202) were used as positive controls, and did not show any significant differences between wt, *mdx* and *tg* serum, whereas miR-302, which specifically expressed in embryonic stem cell, did not detect in all of sera (S1A Fig). In addition, we previously showed that the levels of myomiRs in EVs and EV-depleted supernatant fractions from the sera of DMD patients were significantly increased compared with that of healthy controls [25]. To determine the levels of the three myomiRs in the two fractions from the sera of *tg*, *mdx*, *and* wt mice, sera were separated by centrifugation followed by miRNA extraction. qRT-PCR demonstrated that the levels of the three myomiRs in these fractions were significantly downregulated in *tg* mice compared with *mdx* mice, in which the expression levels of the three myomiRs were significantly increased compared with wt mice (Fig 1A). To analyze the contents of the EVs in the sera of *tg*, *mdx*, and wt mice, EVs were isolated from the sera by centrifugation, and then quantified by AChE activity and EV markers. The amount of EVs, which was verified by exosomal marker proteins (S2A Fig), in the sera of *mdx* and *tg* mice at 7 weeks of age was significantly increased compared with that of wt mice; however, at 13 and 27 weeks of age, there were no significant differences in the amount of EVs between the mice (Fig 1B, left, S2B and S2C Fig). In addition, DMD patients demonstrated significantly greater amounts of EVs compared with healthy controls (Fig 1B, right). Furthermore, to characterize EV markers associated with increases in these miRNA levels, we performed immunoprecipitation assays using five antibodies and EVs from the sera of DMD patients and healthy controls. Levels of three miRNAs (miR-1, miR-133a, and miR-206 levels) and of two miRNAs (miR-1 and miR-133a) were found to be significantly enriched in CD63- and MHC II-associated EVs, respectively, in the serum of DMD patients compared with that of healthy controls (Fig 1C–1E). miR-16 were used as ubiquitous-expressed marker (S3A Fig). Similarly, to identify EV markers in mice, immunoprecipitation was performed using the five antibodies and sera from wt, *mdx*, and *tg* mice. The levels of the three myomiRs were significantly increased in CD63-associated EVs of *mdx* mice compared with wt mice, whereas the levels in *tg* mice were significantly decreased compared with *mdx* mice (S3B Fig). Furthermore, levels of miR-133a and miR-206 were significantly increased in EVs associated with CD81, flotillin-1, and MHC II, and flotillin-1 and MHC II, respectively, in *mdx* mice compared with wt mice (S3B Fig). In addition, miR-128, which expressed specifically in brain, did not detecte in EVs associated with caveolin-3 in wt mice, although miR-128 in other all of EVs were detected (S3B Fig). Ubiquitously expressed miR-16, miR-21, and miR-212 were used as positive controls, and did not show any significant differences between wt, *mdx* and *tg* serum, whereas miR-122a and miR-323 which expressed specifically in liver and brain, respectively, and miR-302 did not dectect in all of EVs (S3B Fig). These results suggest that an increase in the levels of myomiRs in EVs may be associated with muscle degeneration.

### Ceramide metabolic pathways control EV secretion from C_2_C_12_ cells by intracellular calcium-dependent mechanisms

**Fig 2:**
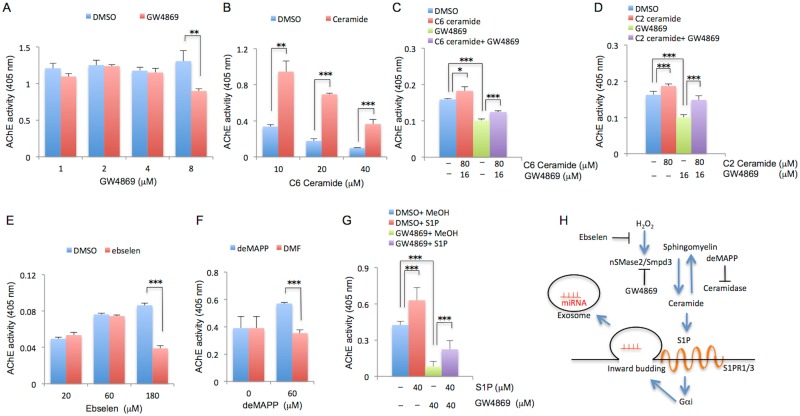
Effects of ceramide and S1P on EV secretion from C_2_C_12_ myoblasts. C_2_C_12_ cells were cultured in growth medium until confluent, and then incubated with serum-depleted medium with or without GW4869 for 72 hr (A), C6-ceramide for 24 hr (B), GW4869 and C6-ceramide (C) or GW4869 and C2-ceramide (D) for 72 hr, ebselen for 48 hr (E), D-erythro-MAPP (deMAPP) for 2 hr (F), and GW4869 or S1P for 48 hr (G). The EVs from these cells were extracted from the culture medium, and the amounts of the released EVs were quantified by measuring AChE activity. Data are represented as means + S.E. of absorbance at 405 nm. *: *P* < 0.05, **: *P* < 0.01, ***: *P* < 0.001. (H) Schematic figure of ceramide biogenesis and metabolism. H_2_O_2_: hydrogen peroxide; S1PR1/3: S1P receptor 1 or 3; Gαi: a subunit of G protein.

Growing lines of evidence have indicated that exosome secretion is regulated by the ceramide synthesizing enzymes nSMase2/Smpd3 or the ceramide-related metabolite, Sphingosine-1-phosphate (S1P) [32, 38]. To assess whether ceramide and S1P control EV secretion in C_2_C_12_ myoblast cells, EVs, confirmed with exosomal protein markers by flow cytometry (S4A Fig) and western blot (S4B Fig), were extracted from the medium of C_2_C_12_ cells cultured with or without the nSMase inhibitor GW4869, and quantified by AChE activity. EV content was significantly decreased by treatment with GW4869 (8 μM) treatment (Fig 2A, *P* < 0.01). In contrast, treatment with C2- or C6-ceramides significantly increased EV release from C_2_C_12_ myoblast cells (Fig 2B–2D), and mitigated the inhibitory effect of EV release by GW4869 (Fig 2C and 2D, *P* < 0.001 and *P* < 0.001, respectively). Moreover, ebselen, which acts as a glutathione peroxidase mimic, significantly reduced the amount of EVs released from C_2_C_12_ myoblast cells (Fig 2E, *P* < 0.001). On the other hand, the ceramidase inhibitor d-erythro-MAPP (60 μM), significantly increased EV release (Fig 2F, *P* < 0.001). Furthermore, S1P (40 uM) significantly increased the release of EVs from C_2_C_12_ myoblast cells (Fig 2G, *P* < 0.001), and ameliorated the inhibitory effect of GW4869 (Fig 2G, *P* < 0.001). These findings taken together suggested that EV release from C_2_C_12_ myoblast cells can be regulated by ceramide metabolism (Fig 2H).

Furthermore, exosome release in some cell types is also known to be regulated by a calcium-dependent mechanism [39–41]. We therefore hypothesized that intracellular calcium also regulates EV secretion in C_2_C_12_ myoblast cells. To test this hypothesis, EVs released from C_2_C_12_ myoblast cells incubated with or without the calcium ionophores monensin and A23187 were quantified by AChE activity. These two calcium ionophores significantly ameliorated the inhibitory effect of GW4869 on EV release from C_2_C_12_ cells (S5A and S5B Fig, *P* < 0.001, *P* < 0.01, respectively). Furthermore, a decrease in EV release by GW4869 was restored by caffeine, a ryanodine receptor activator (S5C Fig). However, the increase in EV release from C_2_C_12_ myoblast cells by d-erythro-MAPP was significantly recovered in the presence of the calcium-chelating agents BAPTA and EGTA (S5D and S5E Fig), or 2-APB, an inhibitor of the inositol 1,4,5-trisphosphate, the InsP3 receptor, and transient receptor potential channels (S5F Fig, *P* < 0.001). Furthermore, increases in EV release by S1P were significantly decreased in the presence of EGTA (S5G Fig, *P* < 0.001). Loperamide, a calcium release-activated channel activator, and cyclopiazonic acid, a sarcoendoplasmic reticulum calcium transport ATPase inhibitor, significantly increased EV release from C_2_C_12_ myoblast cells (S5H and S5I Fig, *P* < 0.001 for each). Taken together, these results indicate that EV release is regulated by intracellular calcium-dependent mechanisms in C_2_C_12_ myoblast cells (S5J Fig).

### Effects of EVs on C_2_C_12_ cell survival

**Fig 3:**
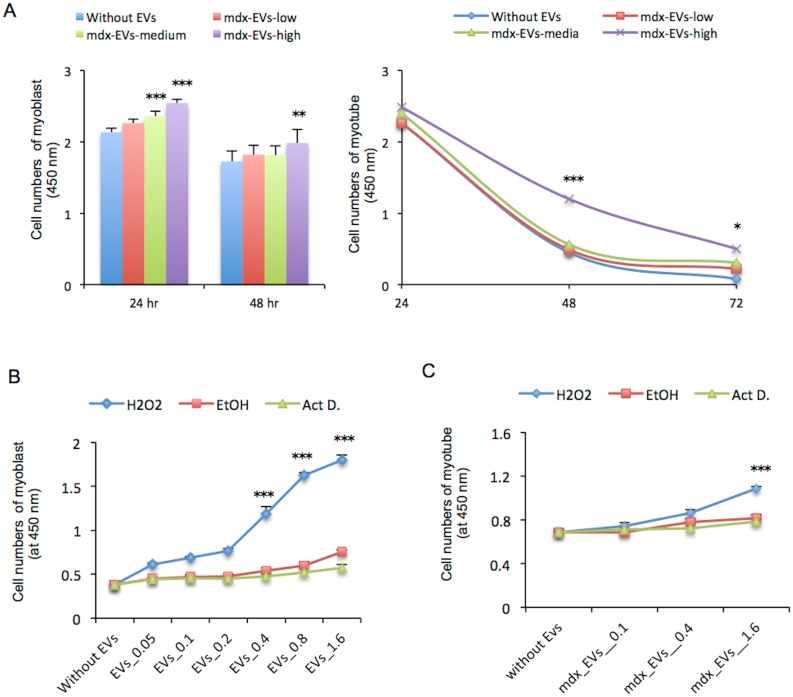
Effect of EVs on the survival of C_2_C_12_ cells. (A) C_2_C_12_ myoblasts (left) and myotubes (right) that were differentiated for 3 days were incubated for the indicated times in serum-depleted medium with low (0.7 μg), medium (2 μg), or high (6 μg) concentrations of EVs that were extracted from the serum of *mdx* mice. (B,C) C_2_C_12_ myoblasts (B) and myotubes (C) differentiated for 6 days were incubated with or without EVs (0.05 μg, 0.1 μg, 0.2 μg, 0.4 μg, 0.8 μg, or 1.6 μg) (B) or (0.7 μg, 2.0 μg, or 6.0 μg) (C) extracted from the serum of mice subjected for 24 hr to three different conditions; H_2_O_2_ (10 mM), ethanol (20%), or actinomycin D (0.5 mg/mL). Data represent mean + S.E. of absorbance at 450 nm of CCK-8. *: *P* < 0.05, **: *P* < 0.01, ***: *P* < 0.001. Each independent experiment was repeated at least 3 times.

Exosomes released from myotubes have been reported to regulate myoblast proliferation and differentiation through intercellular crosstalk [42, 43]. However, the roles of EVs in the muscle degeneration of DMD patients remains to be clarified. To address this issue, C_2_C_12_ cells were incubated in serum-depleted medium with a low (0.7 μg), medium (2 μg), or high (7 μg) concentration of EVs extracted from the serum of *mdx* mice. The survival of myoblasts incubated for 24 hr with a high or medium concentration of EVs extracted from the serum was significantly increased compared with cells incubated without EVs (Fig 3A left, *P* < 0.001, respectively). In addition, the number of myotube cells incubated with a high concentration of EVs for 48 and 72 hrs was significantly increased compared with cells incubated without EVs (Fig 3A right, *P* < 0.001 and *P* < 0.05, respectively). Furthermore, the survival of myoblast cells incubated with a medium or high concentration of EVs extracted from C_2_C_12_ culture medium for 24 hrs was significantly increased compared with that without EVs (S6A Fig left, *P* < 0.05 and *P* < 0.001, respectively). Significant increases in the survival of myotube cells were observed upon incubation with a high concentration of EVs extracted from C_2_C_12_ culture medium for 48 or 72 hrs (S6A Fig right, *P* < 0.001 and *P* < 0.05, respectively). Furthermore, to determine whether EVs regulate the survival of C_2_C_12_ cells in response to apoptosis-associated stress, myoblasts were incubated with EVs from the serum of mice (Fig 3B and 3C) in one of three types of media; with H_2_O_2_, with ethanol, or with actinomycin D. In myoblasts and myotubes, the survival of cells incubated with a high concentration of EVs from the serum of mice was significantly increased in the presence of H_2_O_2_ (Fig 3B and 3C). The uptake of EVs and the mobility of the internalized EVs were reduced by the membrane cholesterol depletion regent methyl-ß-cyclodextrin (MßCD), the microtube polymerization inhibitors, nocodazole and simvastatin, or the MAPK inhibitor U0126 [44]. Increases in the survival of myotubes incubated with EVs from the serum of mice were significantly reversed by the treatment with MßCD, nocodazole, simvastatin, or U0126 (S6B–S6D Fig).

### Effect of myomiRs via EVs on the survival of C_2_C_12_ myoblasts and myotubes

**Fig 4:**
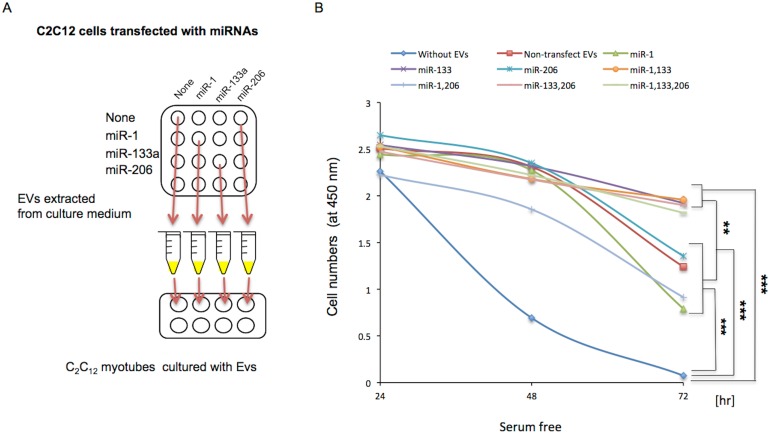
Effect of miRNAs within EVs on the survival of C_2_C_12_ myotubes. (A) Schematic representation of the experiment. C_2_C_12_ cells were cultured and transfected with miR-1, miR-133a, miR-206, miR-1/miR-133a, miR-1/miR-206, miR-133a/miR-206, or miR1/miR-133a/miR-206. Their EVs were extracted from the culture medium, and added to C_2_C_12_ cells in serum-depleted medium. (B) Myotubes differentiated for 4 days were incubated in serum-depleted medium with or without 7 μg of EVs extracted from the medium of C_2_C_12_ cells transfected with miR-1, miR-133a, miR-206, miR-1/miR-133a, miR-1/miR-206, miR-133a/miR-206, or miR-1/miR-133a/miR-206 for the indicated times. Data represent mean + S.E. **: *Pc* < 0.01, ***: *Pc* < 0.001.

Next, to analyze the effect of myomiRs via EVs on cell survival, we performed gain-of-function experiments of miR-1, miR-133a, and miR-206, which were upregulated within EVs during the differentiation of C_2_C_12_ cells (S7 Fig), using C_2_C_12_ cells and EVs extracted from C_2_C_12_ cells transfected with each miRNA (Fig 4A, S8 Fig). The survival of myoblasts incubated with EVs (6 μg) extracted from the culture medium of C_2_C_12_ cells transfected with miR-133a was significantly increased compared with those of cells transfected with miR-1 or miR-206, or untransfected cells in serum-depleted medium (S9A Fig, *Pc* < 0.05). In the presence of H_2_O_2_, the cell survival of myotubes incubated with EVs containing a large amount of miR-133a was significantly increased compared with cells incubated with EVs prepared from untransfected C_2_C_12_ cells (non-TF EVs) (S9B Fig, *P* < 0.05). All of the C_2_C_12_ myotubes cultured with EVs (7 μg) from cells transfected with miR1, miR-133a, or miR-206 demonstrated significantly increased survival compared with cells cultured without EVs in serum-depleted medium for 48 and 72 hr (Fig 4B, *P* < 0.001). Furthermore, we compared the survival of cells treated with two types of EVs, namely, EVs-133a and EVs-non-133a, which are EVs from C_2_C_12_ cells transfected with and without miR-133a, respectively. The cell survival of C_2_C_12_ myotubes cultured with EVs-133a from medium of C_2_C_12_ cells transfected with miR-133a, miR-1/miR-133a, miR-133a/miR-206, or miR-1/miR-133a/miR-206 were significantly increased compared with EVs-non-133a from cells transfected with miR-1, mir-206, miR-1/miR-206, or nontransfected cells in serum-depleted medium for 72 hours (Fig 4B, *P* < 0.01), but not for 24 and 48 hours (Fig 4B). No significant differences were observed in C_2_C_12_ myotubes cultured with EVs from cells transfected with miR-1, miR-206, miR-1/miR-206 compared with that from nontransfected cells (Fig 4B). Furthermore, the numbers of C_2_C_12_ myotubes cultured with low (0.8 μg) or medium (2.0 μg) concentrations of EVs were significantly increased compared with that without EVs for 72 hr (S10A and S10B Fig, *P* < 0.05 and *P* < 0.001, respectively). However, no significant differences were observed in the cell survival of C_2_C_12_ myoblasts cultured with EVs-133a or EVs-non-133a for 48 or 72 hrs (data not shown). These results suggest that the survival of myotubes may be partially regulated by miR-133a via EVs.

### Effect of miR-133a via EVs on the expression of genes associated with apoptosis and differentiation in C_2_C_12_ cells

**Fig 5:**
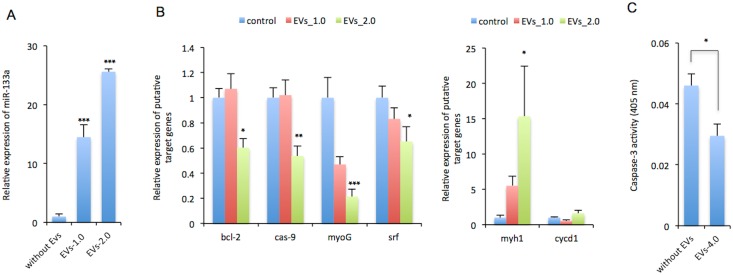
Effect of miRNAs in EVs on C_2_C_12_ cell gene expression. (A) Myotubes were incubated for 72 hr with 1.0 or 2.0 μg of EVs extracted from C_2_C_12_ cells. Total RNA was extracted from the myotubes. Levels of miR-133a (A) and relative mRNA levels of putative target genes (B) were measured by RT-quantitative PCR. (C) Caspase-3 activity was measured in lysates of C_2_C_12_ myotube cells cultured with EVs (4 μg) for 24 hr. Data represent mean + S.E. *: *P* < 0.05, **: *P* < 0.01, ***: *P* < 0.001 versus the relevant control.

We next investigated whether the effect miR-133a via EVs on the survival of myotubes was regulated by its target genes directly or indirectly through the targeting functions of miRNA. To test this hypothesis, we quantified the expression levels of putative target genes in C_2_C_12_ cells cultured with or without EVs containing a large amount of miR-133a. qRT-PCR demonstrated that the levels of miR-133a were significantly upregulated by the incubation of cells with the EVs (Fig 5A). Furthermore, the levels of *bcl-2*, *caspase-9*, *myoG*, and *srf* transcripts were significantly decreased by the incubation of cells with these EVs (Fig 5B, left). Whereas the expression level of *myh1* was significantly upregulated by the incubation of cells with these EVs, *cycd1* was not changed (Fig 5B, right). Moreover, caspase-3 activity in C_2_C_12_ cells cultured with EVs in the presence of H_2_O_2_ was significantly decreased compared with that without EVs (Fig 5C). These findings indicated that the protective effects against apoptosis of C_2_C_12_ cells by EVs might be in part controlled by regulation of the expression of genes targeted by miR-133a.

### Inhibition of ceramide synthesis ameliorates muscular dystrophy in *mdx* mice

**Fig 6:**
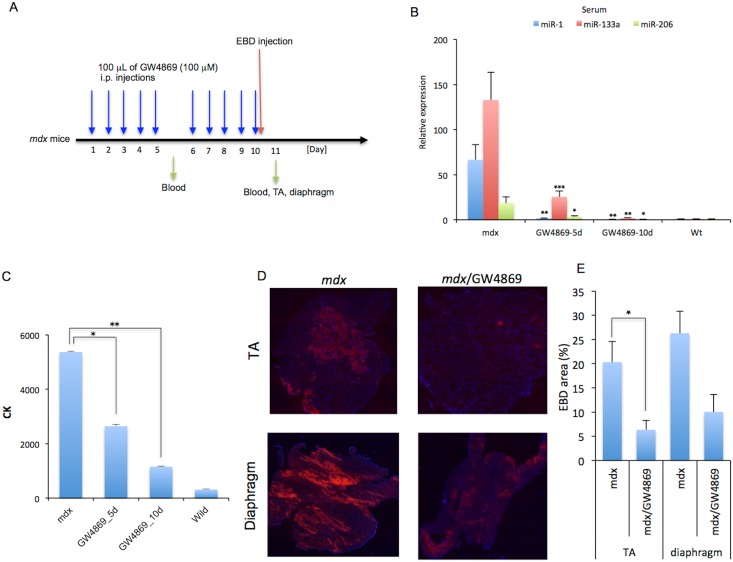
Effects of miRNAs in EVs on muscle regeneration *in vivo*. (A) Experimental timeline of GW4869 administration into *mdx* mice. Six-week old *mdx* mice were injected daily with 100 μL of GW4869 (100 μM) intraperitoneally for 5 or 10 days. After the GW4869 administration period, EBD was injected, and then the next day, whole body blood was collected from the abdominal aorta. miR-1, miR-133a, and miR-206 levels (B) and CK levels (C) in the serum were quantified by RT-quantitative PCR and the Fuji Dri-Chem system, respectively. (D) EBD uptake analyzed in TA muscle and diaphragm sections of *mdx* mice injected with or without GW4869. (E) Quantification of the area of EBD-positive muscle damage in TA muscles and diaphragms of *mdx* mice injected with or without GW4869. Data represent mean + S.E. *: *P* < 0.05, **: *P* < 0.01, ***: *P* < 0.001.

To further investigate whether myomiRs improve muscular dystrophy in *mdx* mice, GW4869 (100 μM) was intraperitoneally administered into *mdx* mice for 5 or 10 days (Fig 6A). After the administration of GW4869, the levels of miR-1, miR-133a, and miR-206, as well as the level of CK, which is indicative of sarcolemmal leakage, were quantified. The levels of myomiRs in the serum (Fig 6B), serum-derived EVs (S11A Fig), and the EV-depleted serum supernatant (S11B Fig) of *mdx* mice treated with GW4869 were significantly decreased compared with that of the controls. Furthermore, serum CK levels of *mdx* mice treated with GW4869 for 5 and 10 days were also significantly decreased compared with those of controls (Fig 6C, *P* < 0.05 and *P* < 0.01, respectively). The tibialis anterior muscle of GW4869-injected *mdx* mice showed a significant decrease in the area of uptake of EBD, which accumulates in damaged cells, compared with that of control *mdx* mice (Fig 6D, top and Fig 6E). On the other hand, only a slight, but insignificant decrease in EBD uptake was observed in the diaphragm of *mdx* mice injected with GW4869 compared with that of the controls (Fig 6D, bottom and Fig 6E). To test the improvement of myofiber structure and function upon GW4869 injection, we analyzed the expression of utrophin in the TA muscle of *mdx* mice treated with GW4869 for 10 days, as well as in untreated *mdx* mice. However, utrophin protein levels in GW4869-treated mice were not increased compared to those of controls (S12 Fig). These results suggested that the inhibition of ceramide synthesis leads to the suppression of EV secretion, which may affect the level of muscular dystrophy in *mdx* mice.

## Discussion

The present study showed that myomiRs are noninvasive biomarkers for the diagnosis of DMD, and that the regulation of myomiR levels may possibly be established as a novel therapy for DMD. We found significant decreases in the levels of miR-1, miR-133a, and miR-206 in the serum of *tg* mice (*mdx* mice overexpressing a truncated dystrophin protein showing normal muscle activities) compared with those of *mdx* mice at 7 weeks of age. Moreover, Roberts et al. reported that restoration of the dystrophin protein by the skipping of exon 23 from the mature dystrophin transcript partially normalized serum myomiRs levels [26]. Furthermore, we previously demonstrated that the upregulation of myomiRs levels in the sera of mice occurred transiently upon CTX-induced injury of their TA one day after the injury [25]. These data suggested that an increase in serum myomiR levels might be dependent on muscle degeneration.

Next, we presented evidence that the secretion of EVs from C_2_C_12_ cells was regulated by sphingolipids metabolism. In agreement with a previous report [45], our study demonstrated significant increases in the secretion of EVs into the extracellular space in the sera of young DMD patients or *mdx* mice compared with controls. However, difference of serum myomiRs level in exosome and exosome-depleted supernatant fractions between our study and previously published data was observed [45]. The two studies were differences for extraction method of exosomes and RNAs from mice serum, and its serum volume as the starting materials. It was reported the difference of number of extracellular vesicles extracted using by Total exosome isolation regent and ultracentrifugation method, although these particle size distributions isolated by the two methods showed no differences and exhibited similar size distribution for exosome, that is a mean diameter of 70–80 nm [46]. Moreover, it was previously reported that the amount of total RNA recovered from exosomes using by Total exosome isolation regent, specifically the small RNA fraction, is higher compared with ultracentrifugation protocol, and the profiles of RNA recovered from exosomes by two different protocol were similar, that is majority of RNA content was < 200 nt [47]. The miRNAs with low GC content or low initial total RNA concentration in the starting materials are recovered inefficiently using by TRIzol compared with mirVana miRNA isolation kit, because of effective isolation of miRNAs needs to base pair with other RNAs as carriers [48]. The GC contents of three myomiRs were relatively low in spite of the unstable secondary structure of the myomiRs based on ΔG values (S1 Table, S13A–S13C Fig). Taken together, the difference of myomiRs level between our present study and published data might be observed. In addition, it was reported that the secretion of myomiRs from cultured C_2_C_12_ myotubes was increased by the addition of serum from DMD patients or the stimulation with fibroblast growth factors (FGF), transforming growth factor-β, or tumor necrosis factor-α (TNF-α) [24]. Furthermore, nSMase2/Smpd3 is activated by TNF-α-stimulated p38 MAPK [49, 50], and preferentially traffics to the plasma membrane upon exposure to an oxidant, such as H_2_O_2_, and is inhibited by the antioxidant, glutathione-SH (GSH) [51, 52]. Thus, the release of EVs into the extracellular space in DMD patients might be induced by the activation of nSMase2/Smpd3, following ceramide production mainly from sphingomyelin in response to some types of cellular stimulation.

In this study, miR-133a in EVs exerted protective effects against C_2_C_12_ cell stress, via the suppression of the expression of apoptosis-associated genes, despite the EVs including various bioactive molecules, such as mRNA, protein, and DNA. It was reported that the overexpression of these myomiRs had little effect on the proliferation or apoptosis of cardiac progenitor cells (CPCs) under basal conditions, whereas miR-133a increased cell survival under oxidative stress in part through targeting of the potent proapoptotic factors, *Bim* and *Bmf* [53]. In addition, overexpression of miR-133a in the skeletal muscle of mice did not result in any overt muscle defects [54]. In our study, C_2_C_12_ cells cultured with EVs in growth medium did not exhibit a significant difference in survival compared with untreated control cells (data not shown). These findings indicate that miR-133a within EVs in the absence of cellular stresses may not affect muscle survival.

In addition, some previous reports indicated that caspase-9 is a possible direct target of miR-133a, and the inhibition of miR-133a increases caspase-9 and caspase-3 activity as well as the number of apoptotic cells in cardiomyocytes [53, 55–57]. Expression levels of caspase-3, caspase-9, and *Bax* expression levels have been shown to be significantly upregulated in the muscle of *mdx* mice [58]. An imbalance between the production and degeneration of myocytes, exacerbated by increased apoptosis and decreased regenerative capacity leads to atrophy in the skeletal muscle of *mdx* mice [59]. Moreover, it was reported that miR-133a is a direct target for apoptotic protease- activating factor 1and has a protective effect on apoptosis by repressing the expression of apoptotic genes [60]. Moreover, miR-133a attenuates hypoxia-induced apoptosis by suppressing caspase-8 signals via its inhibition of the *TAGLN2* gene in cardiomyocytes [61]. It was recently demonstrated that miR-133a inhibits injury-induced cardiomyocyte apoptosis by targeting the *DAPK2* gene [62]. In our study, caspase-3 activity was decreased by miR-133a via EVs in C_2_C_12_ cells under conditions of cell stress. These findings indicate that modification of apoptosis by miR-133a might be a novel therapeutic strategy for DMD.

Expression of the inositol 1,4,5-triphoshate receptor II (IP3RII) calcium channel was constitutively suppressed by miR-133a [55]. In cardiomyocytes, the inhibitory action of miR-133a maintains low basal IP3RII expression. The decline in miR-133a levels resulted in the upregulation of Ca^2+^ levels. In the present study, it was shown that the secretion of EVs from C_2_C_12_ cells may be modulated by intracellular Ca^2+^ concentrations. A chronic increase in Ca^2+^ concentrations in skeletal muscles fibers of DMD patients and *mdx* mice has been observed [63–65]. These reports suggest that the secretion of EVs may be regulated by increases in intracellular Ca^2+^ concentrations, which is induced by IP3RII targeting by miR-133a.

Treatment of *mdx* mice with GW4869 resulted in a significant decrease in myomiR levels in the serum compared with untreated *mdx* mice. In addition, myomiR levels in both EV-containing and EV-depleted supernatant fractions from the sera of GW4869-treated *mdx* mice were significantly lower than in untreated *mdx* mice. It was reported that the local injection of myomiRs or exosomes enhanced muscle regeneration and prevented fibrosis via the expression of myogenic factors in skeletal muscle of injured rat and mouse models [44, 66]. Exosomes extracted from differentiating human myoblasts include various gwoth factors, such as basic FGF, hepatocyte growth factor, insulin like growth factors-I, and vascular endothelial growth factor receptor, which are significantly upregulated in miR-133a-overexpressed adult mouse CPCs [43, 53]. Taking these findings together, the transfer of myomiRs via exosomes may play a role in muscle regeneration, although we cannot exclude the possibility of their beneficial effects on skeletal muscle repair via ceramide or its metabolites.

In summary, our study demonstrated that the release of myomiRs into the blood circulation was associated with the degeneration of skeletal muscle. The secretion of EVs may be regulated by ceramide metabolism by *Smpd3* in C_2_C_12_ cells. Moreover, the transport of miR-133a via EVs into C_2_C_12_ cells increases cell survival under conditions of cellular stress. The inhibition of ceramide synthesis resulted in the suppression of EV secretion, and improved muscle dystrophy in *mdx* mice. Taken together, these findings suggest that EV-mediated myomiR delivery may have potential as a cell-free therapeutic strategy for DMD, by enabling intracellular communication.

## Supporting Information

S1 Fig Levels of myomiRs in the sera of *mdx* mice. (A) Levels of miR-1, miR-133a, and miR-206 in the sera of wt, *mdx*, and *tg* mice (7-weeks old, n = 3, 4, and 4, respectively). miR-21, miR-29, and sno202 are used as ubiquitous-expressed miRNAs. miR-302 is specifically expressed in embryonic stem cell. Data are represented as means + S.E. *: *P* < 0.05, **: *P* < 0.01, ***: *P* < 0.001 for *mdx* vs wt or *mdx* vs *tg*.
(TIFF)

S2 Fig Contents of the EVs from serum. (A) Western blots of exosomes isolated from 200 μL of serum from 5-week old *mdx* mice using antibodies of the exosome markers, CD63, CD9, caveolin-3, and MHC class II. HSP90, apoA-I, and calnexin was used as a positive or negative control for cell lysate from SHSY-5Y cells (cell lysate), exosome and exosome-depleted supernatant (sup). (B)Western blot of Lam2, exosome marker, using 50 μL of serum from 5-weeks old *mdx* or wt mice using antibody against the exosome marker Lamp2. Transferrin was used as a positive control. (C) EV content in the sera of wt, *mdx*, and *tg* mice. EVs were extracted from the sera of wt, *mdx*, and *tg* mice (7, 13, and 27-weeks old) by using Total Exosome Isolation kit. Ten μg/μL of EVs were serially diluted in PBS. One μL of EV solutions (10, 5, 2.5 1.25, 0.63, and 0.31 μg/μL) were subjected to dot blot analysis using anti-MHC class II and anti-CD63 antibodies.
(TIFF)

S3 Fig Levels of miRNAs in EVs. (A) miR-16 level in the EVs separated by immunoprecipitation with anti-caveolin-3 (Cav3), anti-CD63, anti-CD81, anti-flotillin-1 (Flot1), or anti-MHC class II (MHC II) antibodies from the sera of DMD patients and controls (n = 5 and 4, respectively). (B) miR-1, miR-133a, or miR-206 levels in the EVs separated by immunoprecipitation with anti-caveolin-3, anti-CD63, anti-CD81, anti-flotillin-1, or anti-MHC class II antibodies from the sera of wt, *mdx*, and *tg* mice (7-weeks old, n = 3, 4, and 4, respectively). miR-16, miR-21, and miR-212 were used as ubiquitous-expressed miRNAs. miR-122a and miR-323 are specifically expressed in liver and brain, respectively. Data are represented as means + S.E. *: *P* < 0.05, **: *P* < 0.01, ***: *P* < 0.001 for *mdx* vs wt or *mdx* vs *tg*.
(TIFF)

S4 Fig Measurement of the surface expression of caveolin-3, CD9, and CD81 on EVs extracted from the medium of C_2_C_12_ culture cells. (A) EVs covalently bound by aldehyde/sulfate latex beads were mixed with antibodies against caveolin-3, CD9, and CD81, followed by the anti-rat IgG Alexa 488 secondary antibody, and then subjected to flow cytometry analysis. (left) Gating was performed to define area containing EVs, and percentages are indicated. (right) Representative flow cytometric histograms showing caveolin-3, CD9, and CD81-labeled exosome-bead complexes, with percentages indicated for each subpopulation. Staining without secondary antibody was used as a negative control. (B) Western blot analysis of cell lysate and exosomes from C_2_C_12_ myoblast cells using by Total Exosome Isolation Reagent with anti-CD63, anti-caveolin-3, anti-flotillin-1, anti-GAPDH, anti-HSP90, and anti-calnexin antibodies.
(TIFF)

S5 Fig Effects of calcium on the secretion of exosomes from C_2_C_12_ myoblasts. (A-F) C_2_C_12_ cells (90% confluent) were incubated for 24 hr with GW4869 and monensin (A), GW4869 and A23187 (B), or GW4869 and caffeine (C), D-erythro-MAPP and BAPTA (D), D-erythro-MAPP and EGTA (E), or D-erythro-MAPP and 2-APB (F) in serum-depleted medium. (G-I) C_2_C_12_ cells (90% confluent) were incubated for 24 hr with S1P and EGTA (G), loperamide (H), and cyclopiazonic acid (I) in serum-depleted medium. (J) Schematic figure of the effects of calcium on exosome release. CRAC: calcium release-activated channels; Rab: Rab GTPase activating protein; SERCA: sarco/endoplasmic reticulum Ca^2+^-ATPase; RyR: ryanodine receptor; InsP3R: Inositol trisphosphate receptor. The amounts of released exosomes were quantified by measuring AChE activities. Data represent means + S.E. of absorbance at 405 nm. *: *P* < 0.05, **: *P* < 0.01, ***: *P* < 0.001.
(TIFF)

S6 Fig Effect of EVs on C_2_C_12_ cell survival. (A) C_2_C_12_ myoblasts (left) and myotubes (right) were differentiated for 3 days and then incubated for the indicted times in serum-depleted medium with low (0.7 μg), medium (2 μg), or high (6 μg) concentrations of EVs that were extracted from C_2_C_12_ culture medium. (B-D) C_2_C_12_ myoblasts were differentiated for 2 days in 2% serum-containing DMEM, followed by incubation with/without EVs (2 μg) extracted from mouse serum, in serum-free medium with or without 1.0 mM (+) or 2.0 mM (++) of methyl-ß-cyclodextrin (MßCD) for 48 hr (B), or 20 mg/mL of nocodazole (Nocod.) or 2 mM of Simvastatin (Simv.) for 48 hr (C), or 500 mM of U0126 for 24 hr (D). Data are represented as mean + S.D. for absorbance at 450 nm by CCK-8. *: *P* < 0.05, **: *P* < 0.01, ***: *P* < 0.001.
(TIFF)

S7 Fig miR-1, miR-133a, and miR-206 levels within EVs extracted from the culture medium of C_2_C_12_ cells at different stages of differentiation. C_2_C_12_ cells were cultured with growth medium until 90% confluency and then changed to differentiation medium for 1 to 6 days. miRNAs were isolated from EVs extracted from the media of C_2_C_12_ cells on the indicated days and miR-1, miR-133a, and miR-206 levels were measured by RT-quantitative PCR.
(TIFF)

S8 Fig myomiR levels within EVs extracted from C_2_C_12_ cells transfected with each myomiR. miR-1, miR-133a, and miR-206 levels within EVs extracted from the medium of C_2_C_12_ cells transfected with miR-1, miR-133a, or miR-206, and their four possible combinations (miR-1/miR-133a, miR-1/miR-206, miR-133a/miR-206, and miR1/miR-133a/miR-206) were measured by RT-quantitative PCR. Levels are shown relative to that of the non-transfected cells, which was set to 1.
(TIFF)

S9 Fig Survival of myoblasts and myotubes upon incubation with EVs. Myoblasts (A) and myotubes (B), differentiated for 4 days, were incubated with or without low (0.7 μg), medium (2 μg), or high (6 μg) concentrations of EVs extracted from the medium of C_2_C_12_ cells transfected with miR-1, miR-133a, or miR-206, or non-transfected (non-TF EVs) for 24 hrs in serum-depleted medium (A) or in the presence of H_2_O_2_ (10 mM) (B). Data represent mean + S.E. *: *Pc* < 0.05.
(TIFF)

S10 Fig Effect of miRNAs within EVs on C_2_C_12_ myotubes survival. Myotubes were incubated in serum-depleted medium, with 0.8 μg (A), 2 μg (B) of EVs extracted from the medium of C_2_C_12_ cells transfected with miR-1, miR-133a, miR-206, or their four possible combinations (miR-1/miR-133a, miR-1/miR-206, miR-133a/miR-206, and miR1/miR-133a/miR-206) for the indicated times. Data are represented as mean + S.D. for absorbance at 450 nm by CCK-8. *: *P* < 0.05, ***: *P* < 0.001.
(TIFF)

S11 Fig myomiRs levels within EVs or EV-depleted supernatants. miR-1, miR-133a, and miR-206 levels in EVs (A), or EV-depleted supernatants (B) from sera of untreated control *mdx* (*mdx*), GW4869-treated *mdx* for 5 days (GW4869-5d), GW4869-treated *mdx* for 10 days (GW4869-10d), and wt mice were measured by RT-quantitative PCR. Data represent mean + S.E. *: *P* < 0.05, **: *P* < 0.01, ***: *P* < 0.001.
(TIFF)

S12 Fig Expression of utrophin in TA muscle. TA muscle lysates of *mdx* mice treated with GW4869 for 10 days or untreated control *mdx* mice were subjected to western blotting to analyze utrophin expression. Gapdh (Glyceraldehyde 3-phosphate dehydrogenase) and tublin were used as loading controls.
(TIFF)

S13 Fig Secondary structure of the myomiRs, miR-1 (A), miR-133a (B), and miR-206 (C) based on ΔG values. (TIFF)

S1 Table Secondary structure of myomiRs. GC contents and ΔG for mature sequences of myomiRs were estimated.
(XLSX)
