# Supplementary figures and images for: Characterization and Functional Analysis of Extracellular Vesicles and Muscle-Abundant miRNAs (miR-1, miR-133a, and miR-206) in C2C12 Myocytes and mdx Mice
Source: PLoS One. 2016 Dec 15;11(12):e0167811. doi: 10.1371/journal.pone.0167811 (PMC5158003; doi:10.1371/journal.pone.0167811)

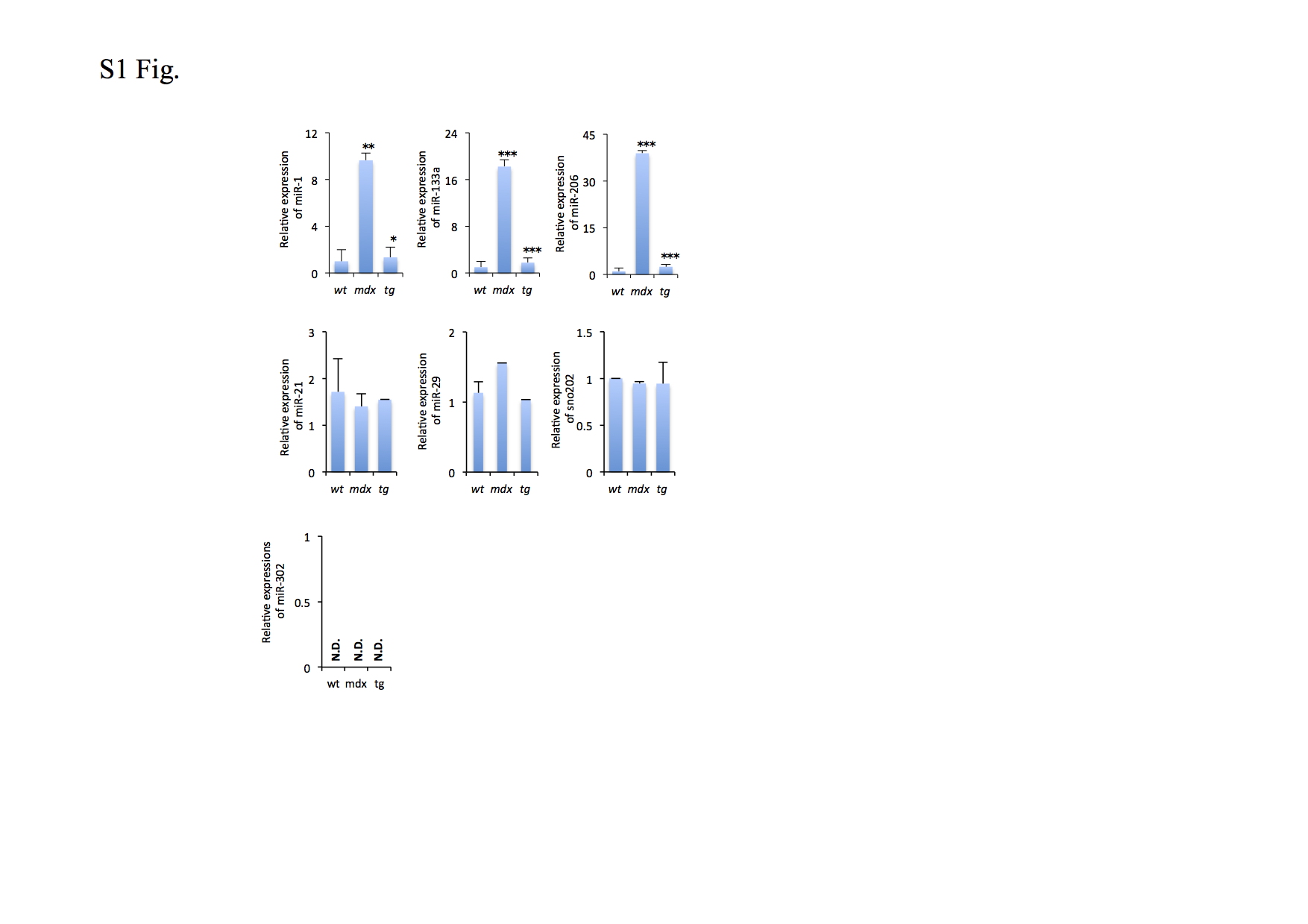

Supplement: S1 Fig — (A) Levels of miR-1, miR-133a, and miR-206 in the sera of wt, mdx, and tg mice (7-weeks old, n = 3, 4, and 4, respectively). miR-21, miR-29, and sno202 are used as ubiquitous-expressed miRNAs. miR-302 is specifically expressed in embryonic stem cell. Data are represented as means + S.E. *: P < 0.05, **: P < 0.01, ***: P < 0.001 for mdx vs wt or mdx vs tg. (TIFF) [file pone.0167811.s001.tiff]

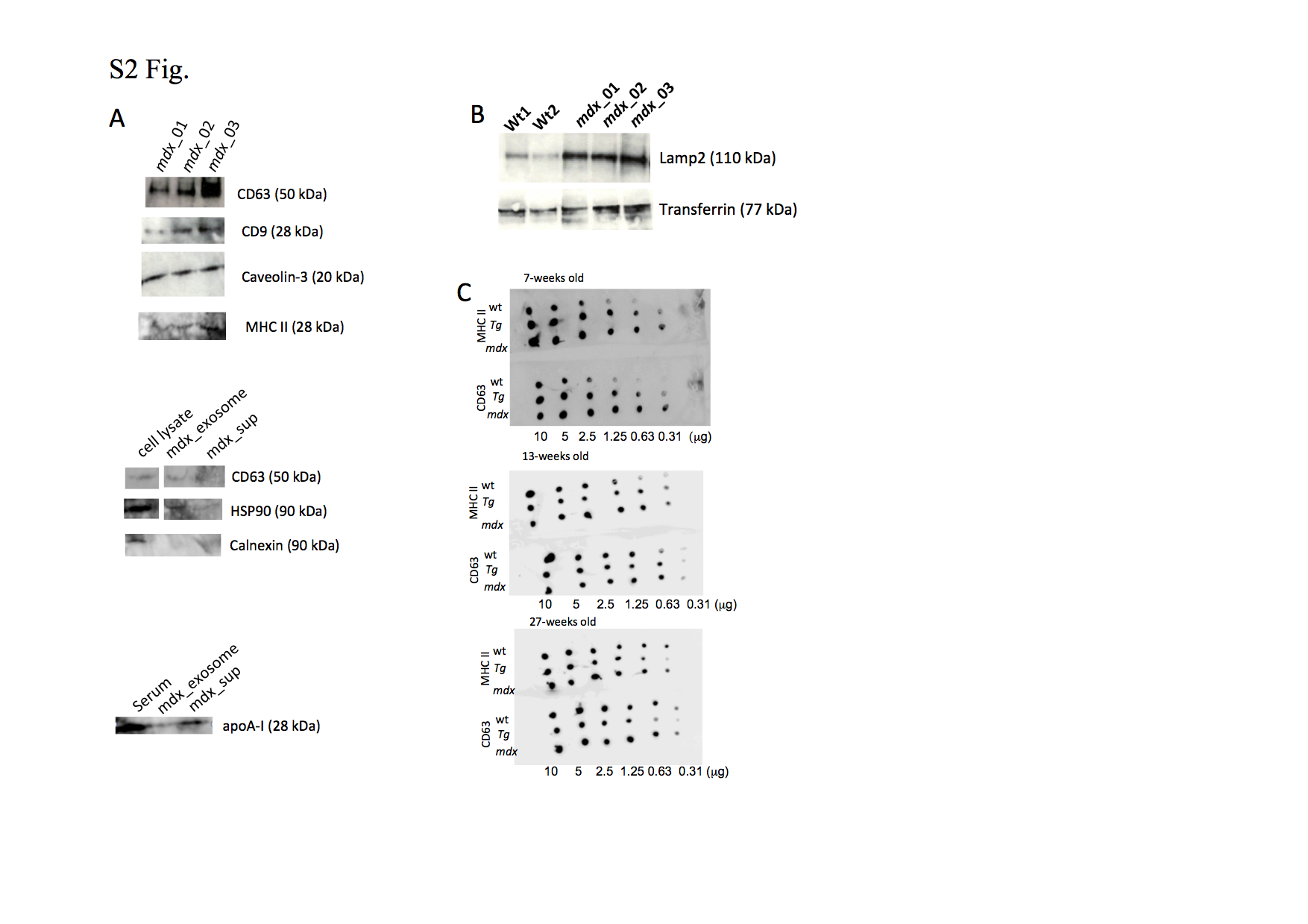

Supplement: S2 Fig — (A) Western blots of exosomes isolated from 200 μL of serum from 5-week old mdx mice using antibodies of the exosome markers, CD63, CD9, caveolin-3, and MHC class II. HSP90, apoA-I, and calnexin was used as a positive or negative control for cell lysate from SHSY-5Y cells (cell lysate), exosome and exosome-depleted supernatant (sup). (B)Western blot of Lam2, exosome marker, using 50 μL of serum from 5-weeks old mdx or wt mice using antibody against the exosome marker Lamp2. Transferrin was used as a positive control. (C) EV content in the sera of wt, mdx, and tg mice. EVs were extracted from the sera of wt, mdx, and tg mice (7, 13, and 27-weeks old) by using Total Exosome Isolation kit. Ten μg/μL of EVs were serially diluted in PBS. One μL of EV solutions (10, 5, 2.5 1.25, 0.63, and 0.31 μg/μL) were subjected to dot blot analysis using anti-MHC class II and anti-CD63 antibodies. (TIFF) [file pone.0167811.s002.tiff]

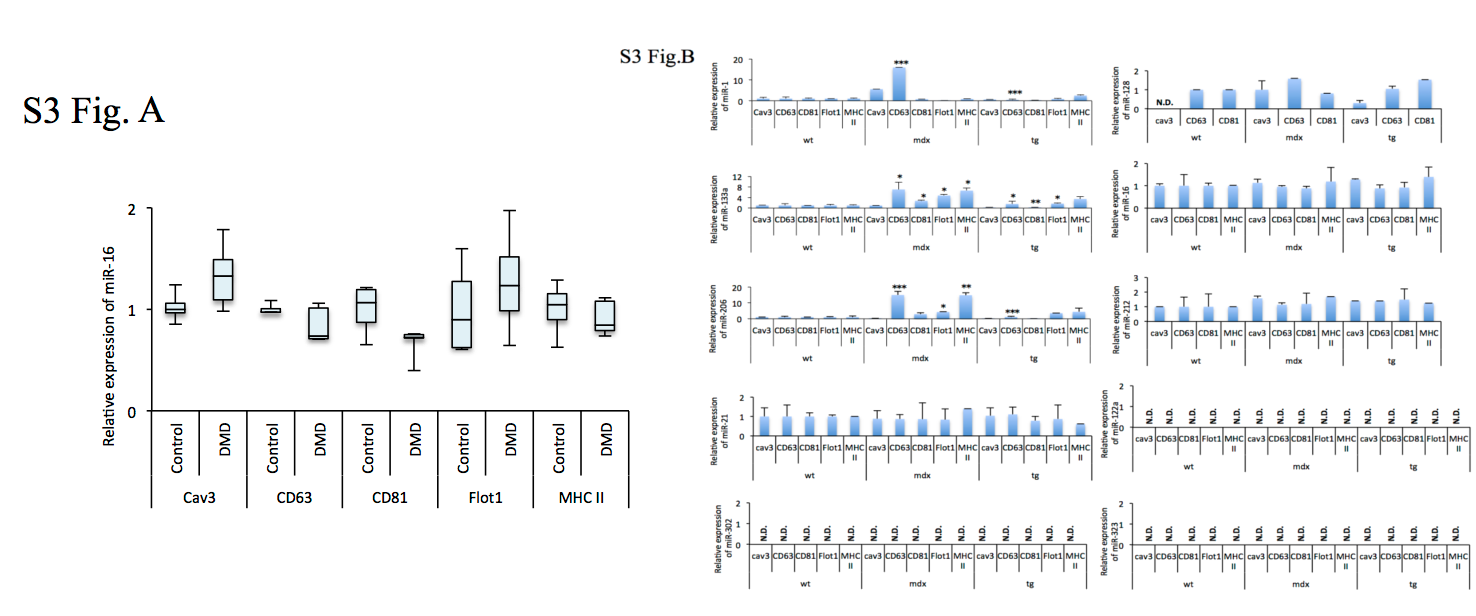

Supplement: S3 Fig — (A) miR-16 level in the EVs separated by immunoprecipitation with anti-caveolin-3 (Cav3), anti-CD63, anti-CD81, anti-flotillin-1 (Flot1), or anti-MHC class II (MHC II) antibodies from the sera of DMD patients and controls (n = 5 and 4, respectively). (B) miR-1, miR-133a, or miR-206 levels in the EVs separated by immunoprecipitation with anti-caveolin-3, anti-CD63, anti-CD81, anti-flotillin-1, or anti-MHC class II antibodies from the sera of wt, mdx, and tg mice (7-weeks old, n = 3, 4, and 4, respectively). miR-16, miR-21, and miR-212 were used as ubiquitous-expressed miRNAs. miR-122a and miR-323 are specifically expressed in liver and brain, respectively. Data are represented as means + S.E. *: P < 0.05, **: P < 0.01, ***: P < 0.001 for mdx vs wt or mdx vs tg. (TIFF) [file pone.0167811.s003.tiff]

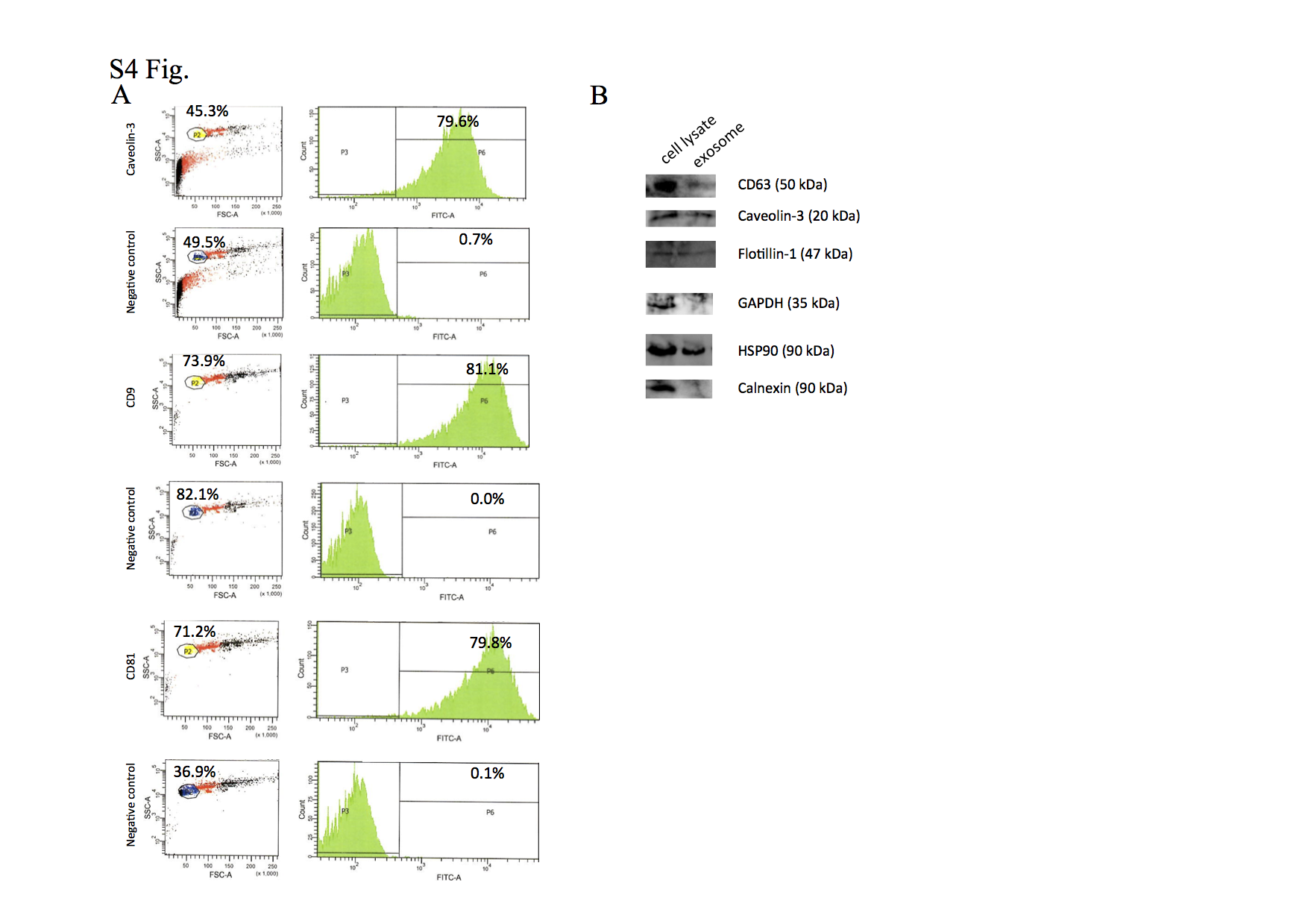

Supplement: S4 Fig — (A) EVs covalently bound by aldehyde/sulfate latex beads were mixed with antibodies against caveolin-3, CD9, and CD81, followed by the anti-rat IgG Alexa 488 secondary antibody, and then subjected to flow cytometry analysis. (left) Gating was performed to define area containing EVs, and percentages are indicated. (right) Representative flow cytometric histograms showing caveolin-3, CD9, and CD81-labeled exosome-bead complexes, with percentages indicated for each subpopulation. Staining without secondary antibody was used as a negative control. (B) Western blot analysis of cell lysate and exosomes from C2C12 myoblast cells using by Total Exosome Isolation Reagent with anti-CD63, anti-caveolin-3, anti-flotillin-1, anti-GAPDH, anti-HSP90, and anti-calnexin antibodies. (TIFF) [file pone.0167811.s004.tiff]

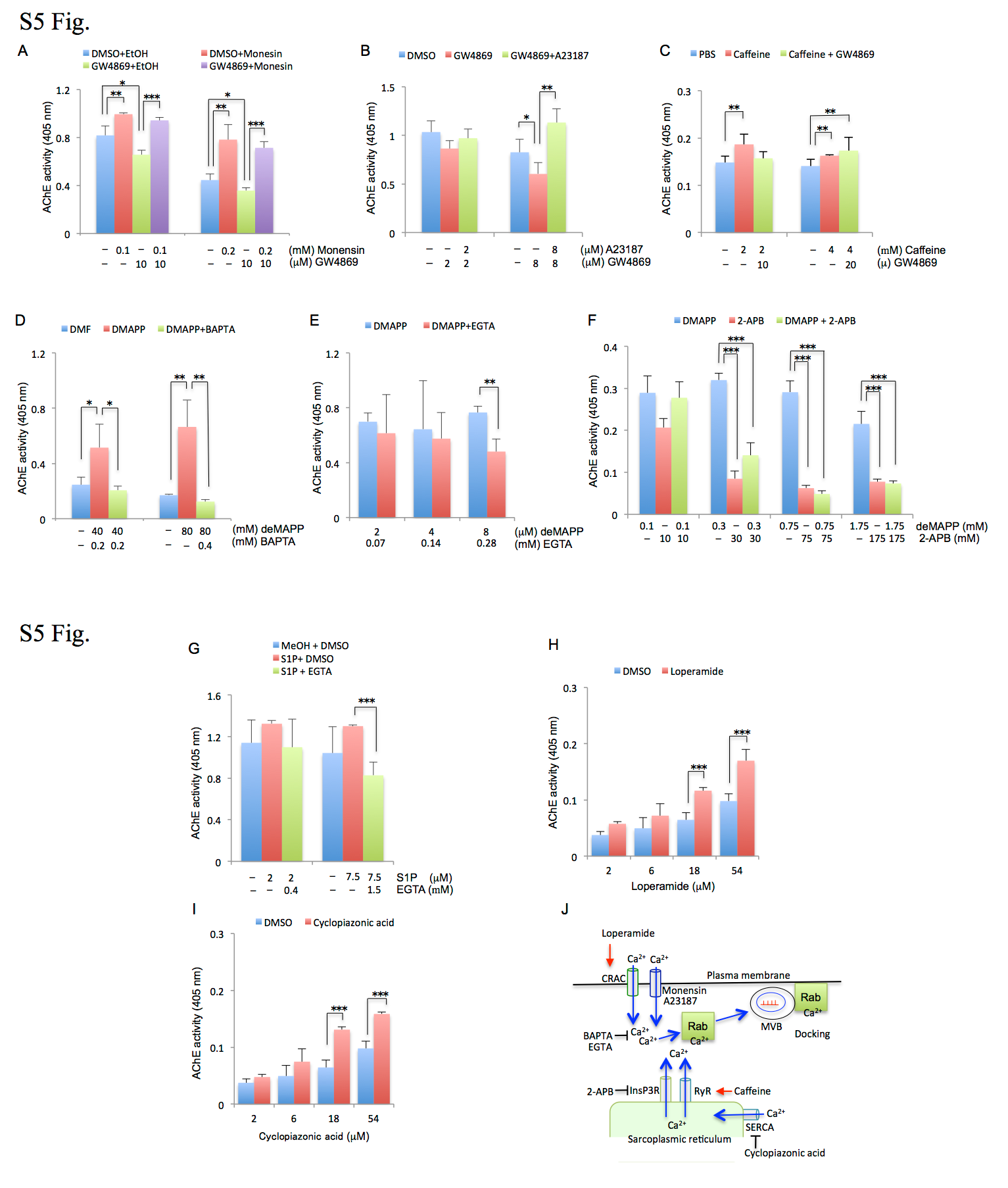

Supplement: S5 Fig — (A-F) C2C12 cells (90% confluent) were incubated for 24 hr with GW4869 and monensin (A), GW4869 and A23187 (B), or GW4869 and caffeine (C), D-erythro-MAPP and BAPTA (D), D-erythro-MAPP and EGTA (E), or D-erythro-MAPP and 2-APB (F) in serum-depleted medium. (G-I) C2C12 cells (90% confluent) were incubated for 24 hr with S1P and EGTA (G), loperamide (H), and cyclopiazonic acid (I) in serum-depleted medium. (J) Schematic figure of the effects of calcium on exosome release. CRAC: calcium release-activated channels; Rab: Rab GTPase activating protein; SERCA: sarco/endoplasmic reticulum Ca2+-ATPase; RyR: ryanodine receptor; InsP3R: Inositol trisphosphate receptor. The amounts of released exosomes were quantified by measuring AChE activities. Data represent means + S.E. of absorbance at 405 nm. *: P < 0.05, **: P < 0.01, ***: P < 0.001. (TIFF) [file pone.0167811.s005.tiff]

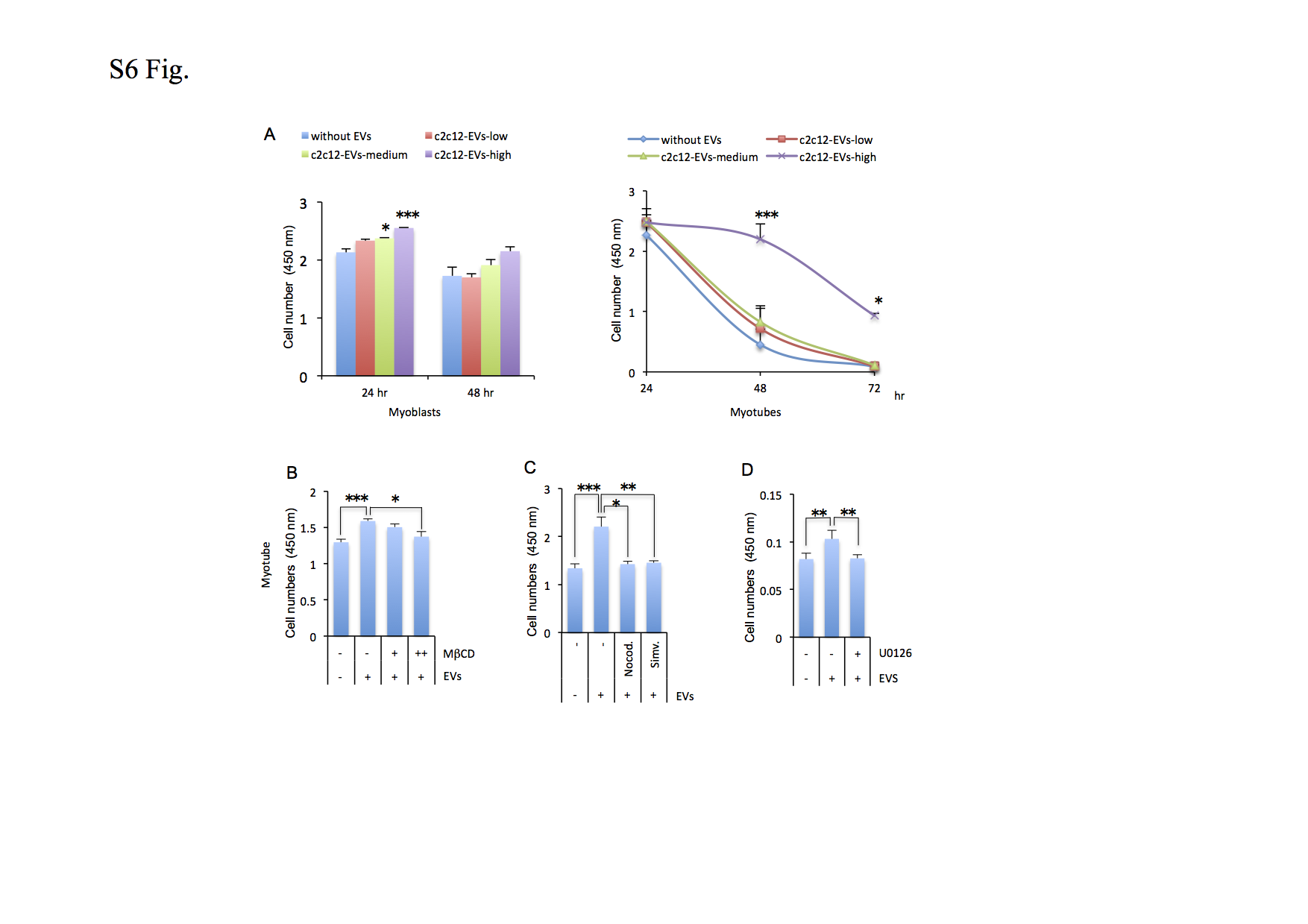

Supplement: S6 Fig — (A) C2C12 myoblasts (left) and myotubes (right) were differentiated for 3 days and then incubated for the indicted times in serum-depleted medium with low (0.7 μg), medium (2 μg), or high (6 μg) concentrations of EVs that were extracted from C2C12 culture medium. (B-D) C2C12 myoblasts were differentiated for 2 days in 2% serum-containing DMEM, followed by incubation with/without EVs (2 μg) extracted from mouse serum, in serum-free medium with or without 1.0 mM (+) or 2.0 mM (++) of methyl-ß-cyclodextrin (MßCD) for 48 hr (B), or 20 mg/mL of nocodazole (Nocod.) or 2 mM of Simvastatin (Simv.) for 48 hr (C), or 500 mM of U0126 for 24 hr (D). Data are represented as mean + S.D. for absorbance at 450 nm by CCK-8. *: P < 0.05, **: P < 0.01, ***: P < 0.001. (TIFF) [file pone.0167811.s006.tiff]

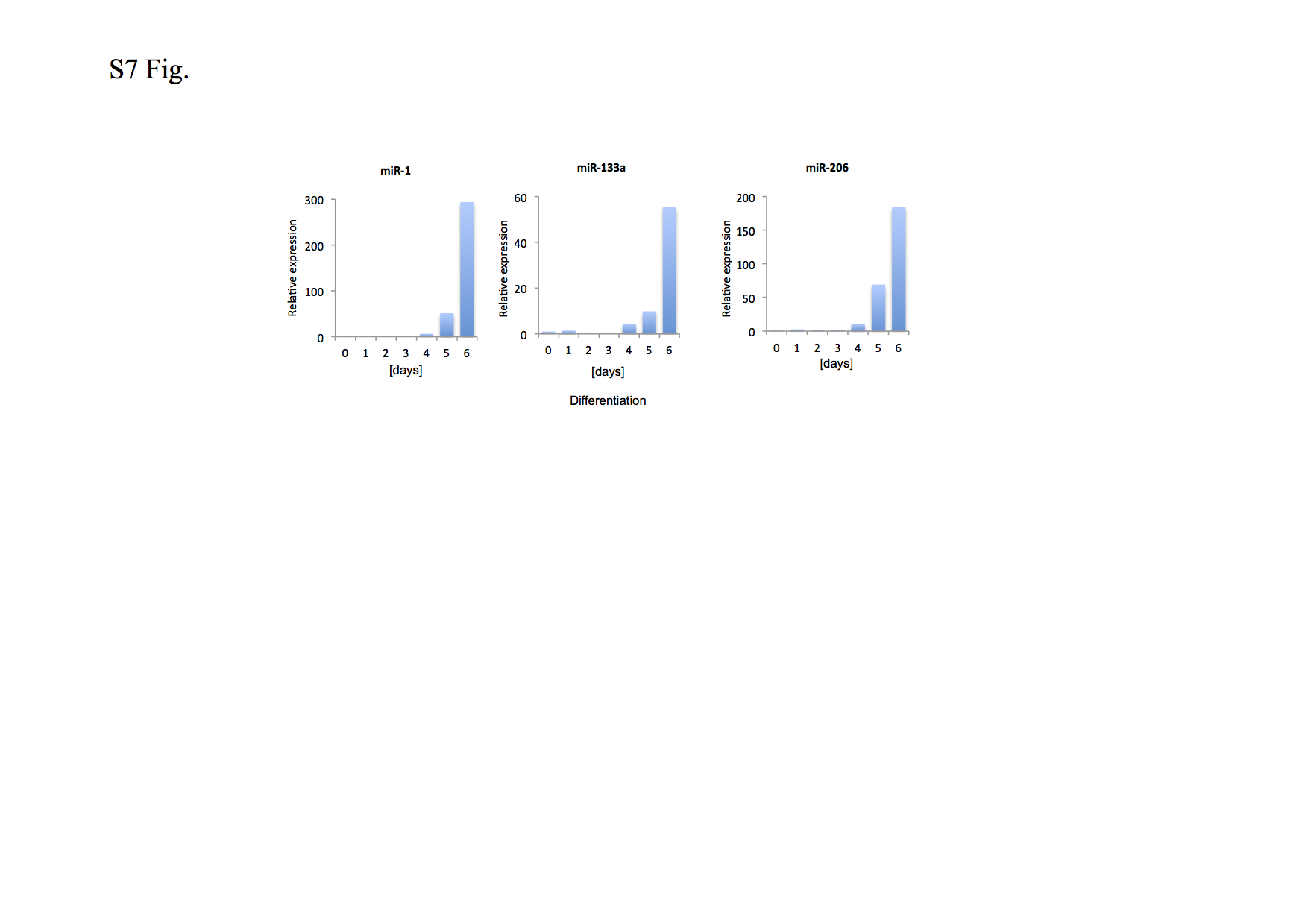

Supplement: S7 Fig — C2C12 cells were cultured with growth medium until 90% confluency and then changed to differentiation medium for 1 to 6 days. miRNAs were isolated from EVs extracted from the media of C2C12 cells on the indicated days and miR-1, miR-133a, and miR-206 levels were measured by RT-quantitative PCR. (TIFF) [file pone.0167811.s007.tiff]

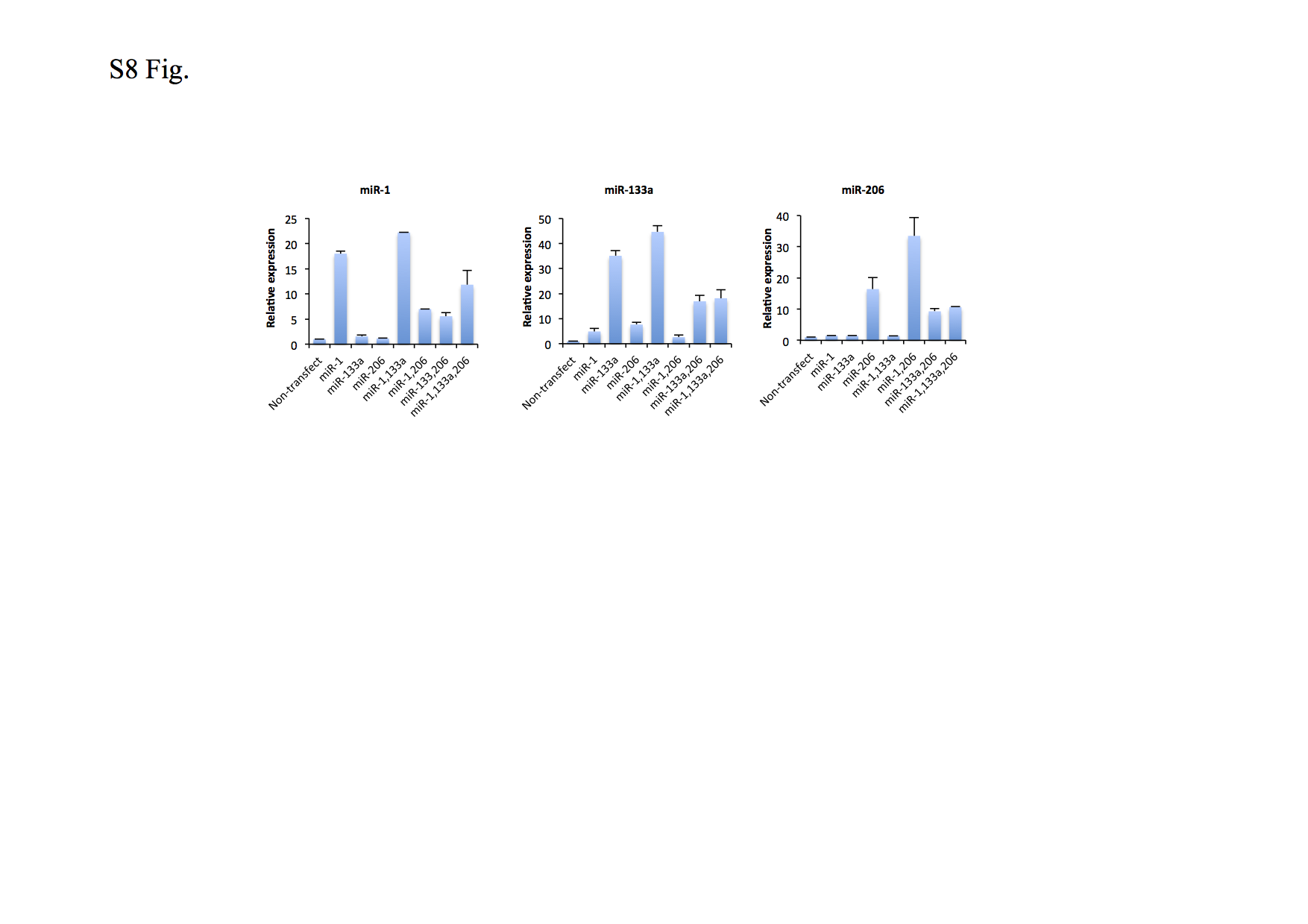

Supplement: S8 Fig — miR-1, miR-133a, and miR-206 levels within EVs extracted from the medium of C2C12 cells transfected with miR-1, miR-133a, or miR-206, and their four possible combinations (miR-1/miR-133a, miR-1/miR-206, miR-133a/miR-206, and miR1/miR-133a/miR-206) were measured by RT-quantitative PCR. Levels are shown relative to that of the non-transfected cells, which was set to 1. (TIFF) [file pone.0167811.s008.tiff]

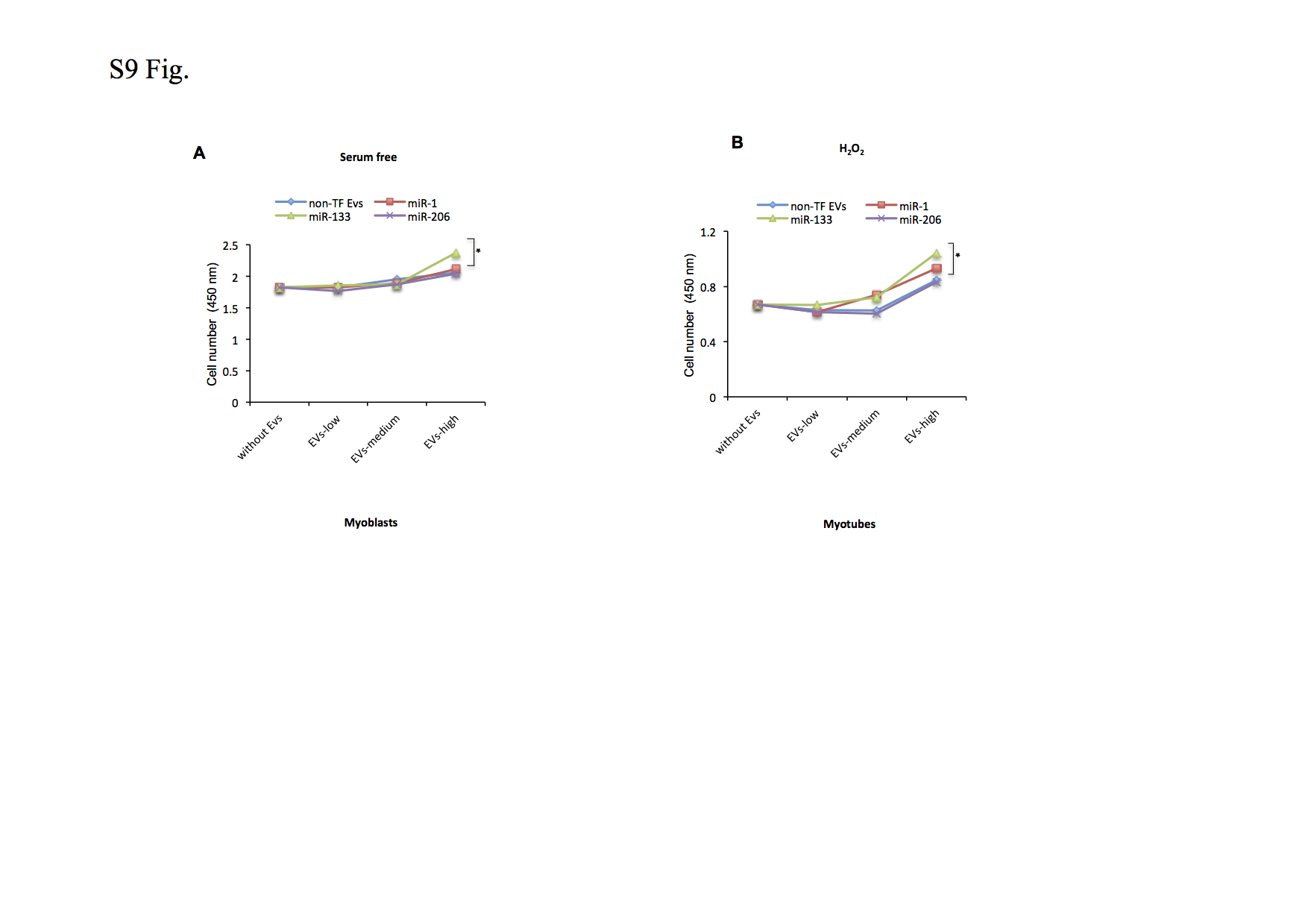

Supplement: S9 Fig — Myoblasts (A) and myotubes (B), differentiated for 4 days, were incubated with or without low (0.7 μg), medium (2 μg), or high (6 μg) concentrations of EVs extracted from the medium of C2C12 cells transfected with miR-1, miR-133a, or miR-206, or non-transfected (non-TF EVs) for 24 hrs in serum-depleted medium (A) or in the presence of H2O2 (10 mM) (B). Data represent mean + S.E. *: Pc < 0.05. (TIFF) [file pone.0167811.s009.tiff]

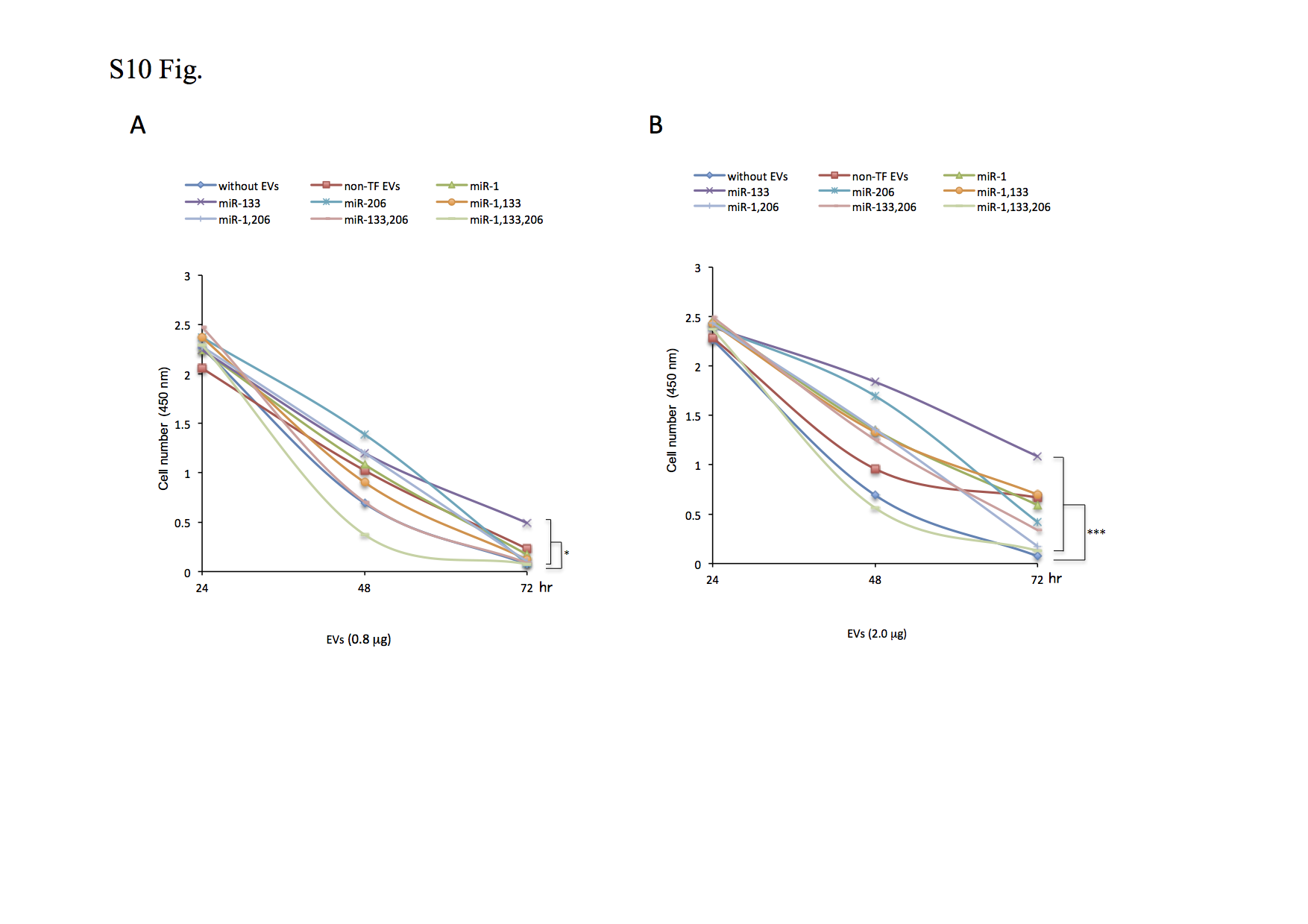

Supplement: S10 Fig — Myotubes were incubated in serum-depleted medium, with 0.8 μg (A), 2 μg (B) of EVs extracted from the medium of C2C12 cells transfected with miR-1, miR-133a, miR-206, or their four possible combinations (miR-1/miR-133a, miR-1/miR-206, miR-133a/miR-206, and miR1/miR-133a/miR-206) for the indicated times. Data are represented as mean + S.D. for absorbance at 450 nm by CCK-8. *: P < 0.05, ***: P < 0.001. (TIFF) [file pone.0167811.s010.tiff]

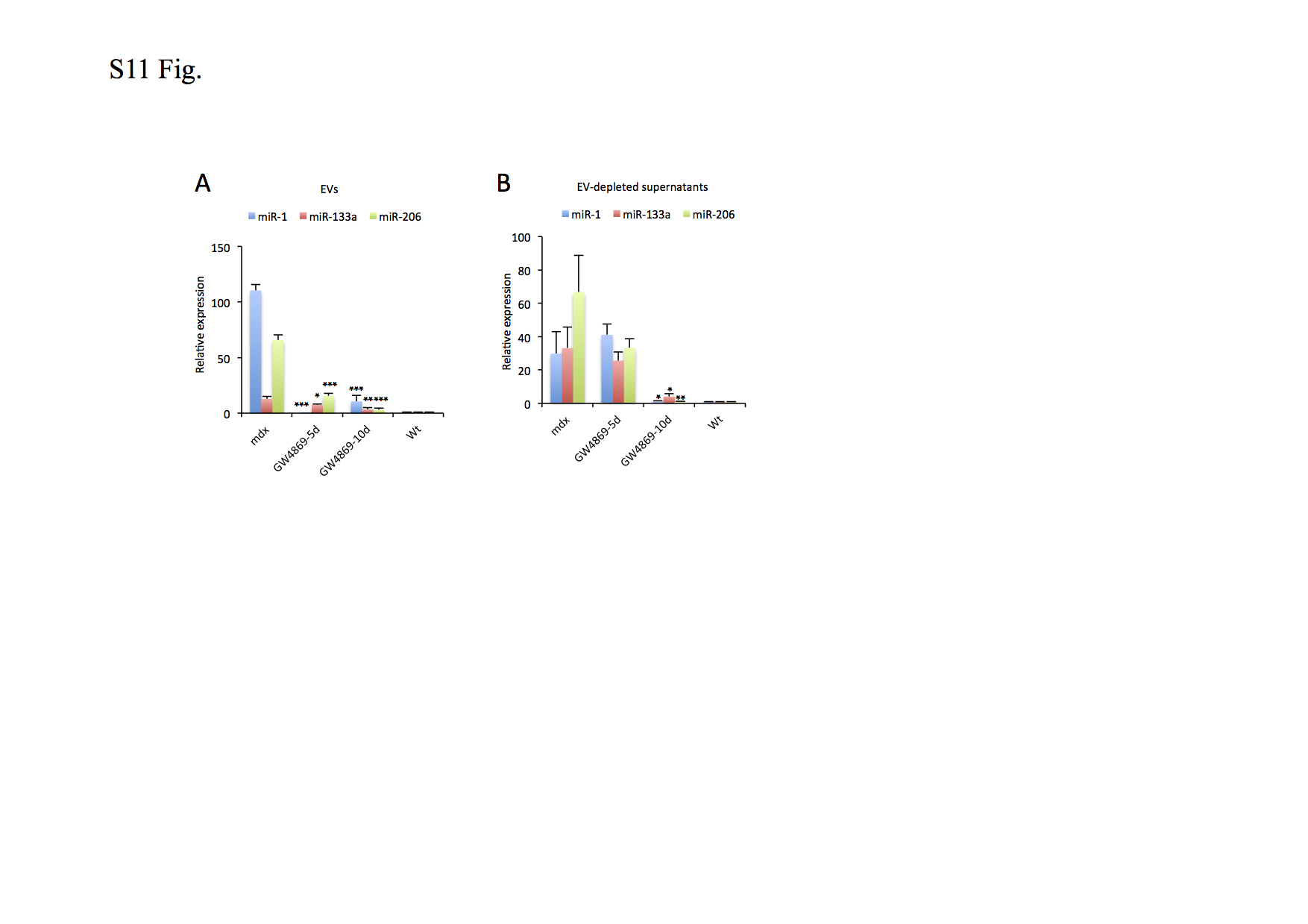

Supplement: S11 Fig — miR-1, miR-133a, and miR-206 levels in EVs (A), or EV-depleted supernatants (B) from sera of untreated control mdx (mdx), GW4869-treated mdx for 5 days (GW4869-5d), GW4869-treated mdx for 10 days (GW4869-10d), and wt mice were measured by RT-quantitative PCR. Data represent mean + S.E. *: P < 0.05, **: P < 0.01, ***: P < 0.001. (TIFF) [file pone.0167811.s011.tiff]

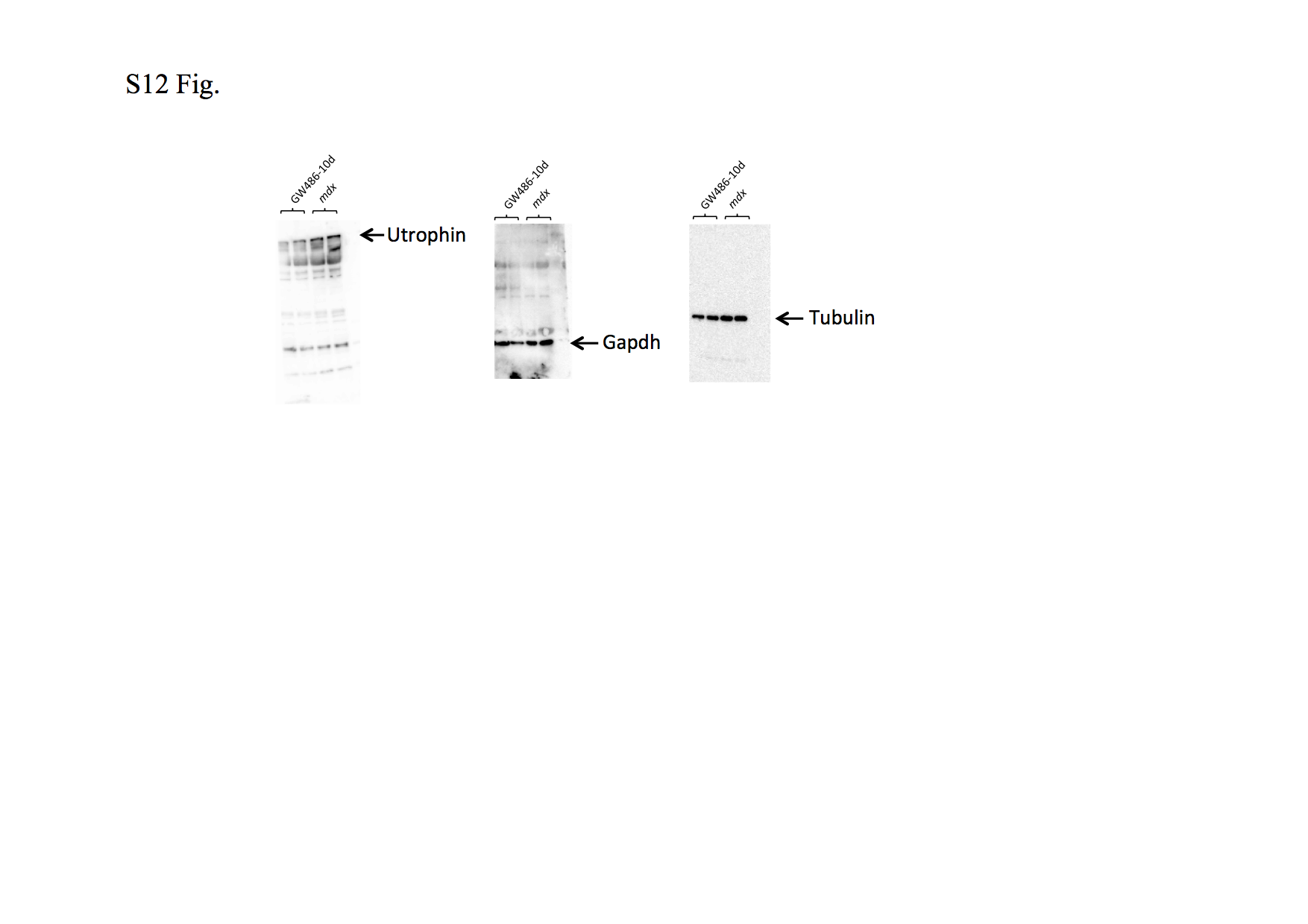

Supplement: S12 Fig — TA muscle lysates of mdx mice treated with GW4869 for 10 days or untreated control mdx mice were subjected to western blotting to analyze utrophin expression. Gapdh (Glyceraldehyde 3-phosphate dehydrogenase) and tublin were used as loading controls. (TIFF) [file pone.0167811.s012.tiff]

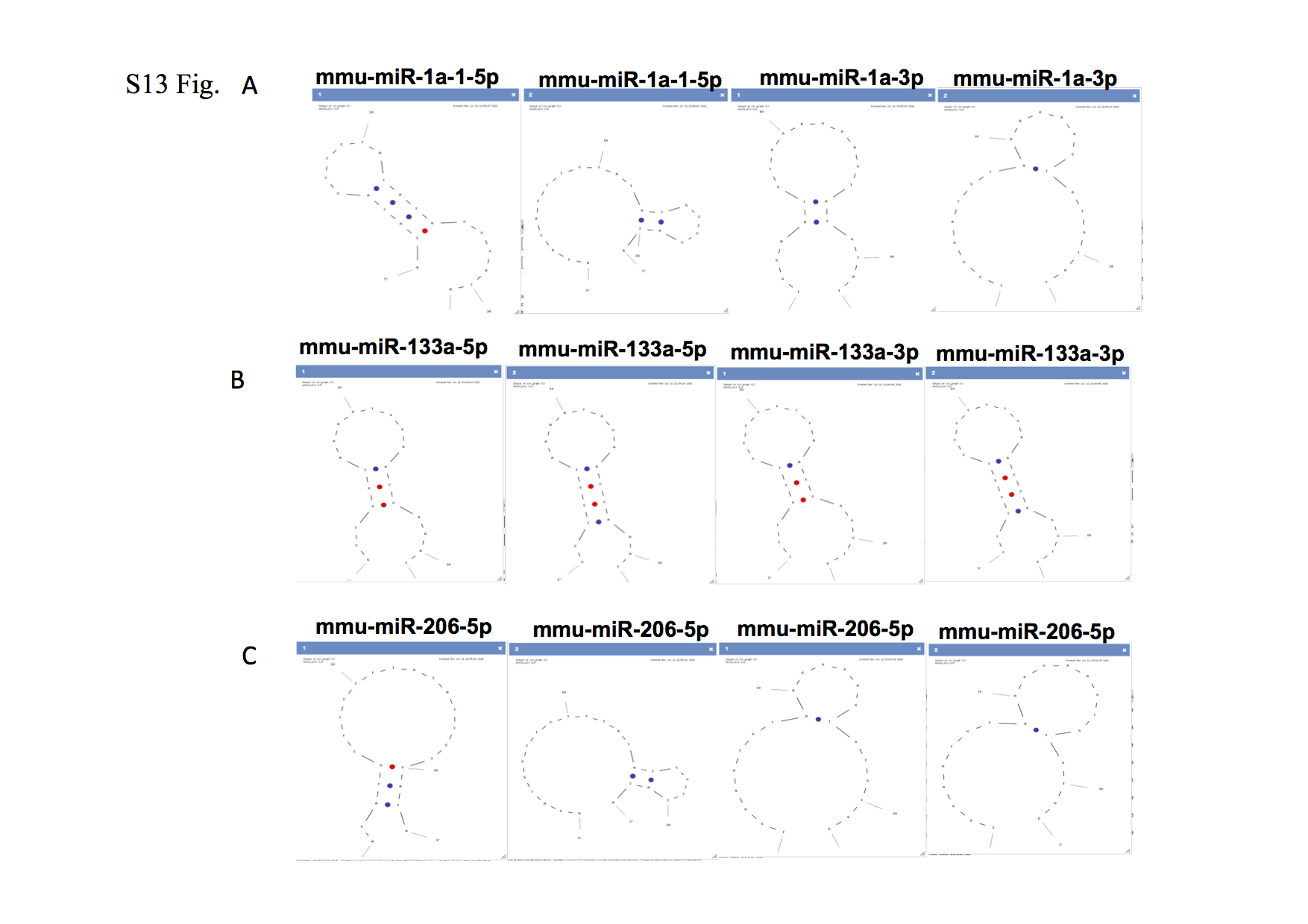

Supplement: S13 Fig — (TIFF) [file pone.0167811.s013.tiff]
